# Targeting NAD^+^ Metabolism in the Human Malaria Parasite *Plasmodium falciparum*

**DOI:** 10.1371/journal.pone.0094061

**Published:** 2014-04-18

**Authors:** Jessica K. O'Hara, Lewis J. Kerwin, Simon A. Cobbold, Jonathan Tai, Thomas A. Bedell, Paul J. Reider, Manuel Llinás

**Affiliations:** 1 Department of Molecular Biology and Lewis-Sigler Institute for Integrative Genomics, Princeton University, Princeton, New Jersey, United States of America; 2 Department of Chemistry, Princeton University, Princeton, New Jersey, United States of America; 3 Department of Biochemistry, University of Illinois at Urbana-Champaign, Urbana, Illinois, United States of America; University of Hull, United Kingdom

## Abstract

Nicotinamide adenine dinucleotide (NAD^+^) is an essential metabolite utilized as a redox cofactor and enzyme substrate in numerous cellular processes. Elevated NAD^+^ levels have been observed in red blood cells infected with the malaria parasite *Plasmodium falciparum*, but little is known regarding how the parasite generates NAD^+^. Here, we employed a mass spectrometry-based metabolomic approach to confirm that *P. falciparum* lacks the ability to synthesize NAD^+^ *de novo* and is reliant on the uptake of exogenous niacin. We characterized several enzymes in the NAD^+^ pathway and demonstrate cytoplasmic localization for all except the parasite nicotinamidase, which concentrates in the nucleus. One of these enzymes, the *P. falciparum* nicotinate mononucleotide adenylyltransferase (PfNMNAT), is essential for NAD^+^ metabolism and is highly diverged from the human homolog, but genetically similar to bacterial NMNATs. Our results demonstrate the enzymatic activity of PfNMNAT *in vitro* and demonstrate its ability to genetically complement the closely related *Escherichia coli* NMNAT. Due to the similarity of PfNMNAT to the bacterial enzyme, we tested a panel of previously identified bacterial NMNAT inhibitors and synthesized and screened twenty new derivatives, which demonstrate a range of potency against live parasite culture. These results highlight the importance of the parasite NAD^+^ metabolic pathway and provide both novel therapeutic targets and promising lead antimalarial compounds.

## Introduction

Malaria remains one of the most devastating and prevalent infectious diseases worldwide, with 350 to 500 million annual cases, imposing a heavy burden on the healthcare and economic development of afflicted countries [1], [2]. The Apicomplexan parasite *Plasmodium falciparum* is responsible for the most severe form of malaria killing 650,000 individuals in 2011, with 86% of deaths occurring in children under the age of five [3]. The recent rise in drug resistant parasite strains has increased the burden of malaria and drawn attention to the need for the identification of novel drug targets and new antimalarial therapeutics. Many of the clinical symptoms of malaria are tied to the metabolic stresses placed on the host when the parasite infects and develops within the red blood cell. As the parasite rapidly grows and divides during its 48 hour asexual life cycle it is greatly dependent on glycolysis for energy production. Plasmodium-infected erythrocytes can consume glucose at approximately one hundred times the rate of uninfected erythrocytes [4], [5]. This primary reliance on anaerobic respiration is coincidental with some of the most distinguishable clinical symptoms associated with malaria, such as hypoglycemia and lactic acidosis. Therefore, increased characterization of the poorly understood metabolism of *P. falciparum* is important to understand many of the host-parasite interactions that underlie the clinical symptoms of malaria and for identifying both novel pathways and specific enzymes to target therapeutically.

Studies in four different *Plasmodium* species have previously reported that NAD^+^ levels are high during the asexual blood stage of development, with infected erythrocytes exhibiting 5 to 10-fold higher concentrations compared to uninfected red blood cells [6]–[9]. NAD^+^ and its phosphorylated (NADP^+^) and reduced forms (NADH and NADPH) are essential to the central metabolism of all organisms and are well understood for their role as important redox cofactors [10]. In recent years, however, NAD^+^ has gained recognition for its diverse role as an enzyme substrate in a number of essential cellular processes including epigenetic regulation, calcium signaling, and DNA repair [11], [12]. The *P. falciparum* genome appears to encode significantly fewer NAD^+^ utilizing enzymes than other organisms, containing only two putative sirtuin proteins (Sir2) and no homologs of poly(ADP-ribose) polymerase [13]. Sir2 proteins catalyze the deacetylation of proteins, most notably histones, in a NAD^+^-dependent manner [14]. In *P. falciparum* the two Sir2 proteins (PF13_0152, PfSIR2A and PF14_0489, PfSIR2B) are involved in telomere maintenance and genetic regulation of the subtelomeric *var* gene family, which encodes the *P. falciparum* erythrocyte membrane protein 1 (PfEMP1) surface protein, an important factor in parasite cytoadherence and virulence [15]–[18]. Due to the catabolic NAD^+^ requirement by the Sir2s [14], and the requirement of NAD^+^ as a cofactor for many other NAD^+^ dependent enzymes, it is likely that regulation of the NAD^+^ metabolic pathway provides a link between metabolism and a variety of important cellular processes in the *Plasmodium* parasite.

**Figure 1:**
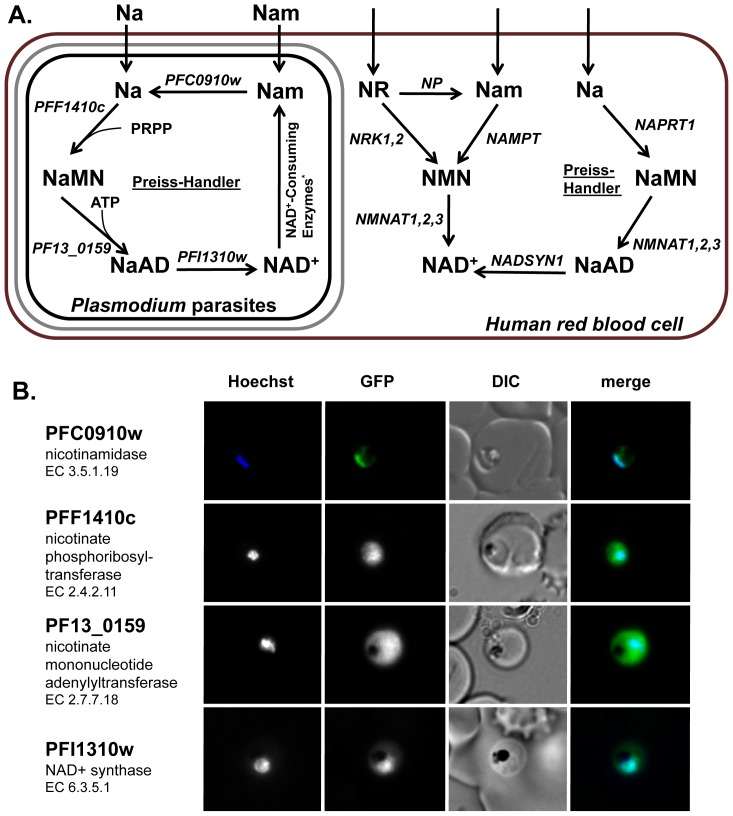
*Plasmodium falciparum* NAD^+^ metabolism. **A**. Overview of the NAD^+^ salvage pathways in *Plasmodium* parasites and the human host red blood cell. Plasmodium falciparum gene IDs and mammalian gene names are presented for each enzyme in italics. The red blood cell is shown in red, the parasitophorous membrane is shown in grey and the parasite membrane is shown in black. Na: nicotinic acid; Nam: nicotinamide; NR: nicotinamide ribotide; NaMN: nicotinate mononucleotide; NMN: nicotinamide mononucleotide; NaAD: nicotinate adenine dinucleotide; NAD^+^: nicotinamide adenine dinucleotide. NAPRT: nicotinic acid phosphoribosyltransferase, NMNAT: mononucleotide adenylyl transferase, NRK: nicotinamide riboside kinase, NP: nucleoside phosphorylase, NAMPT: nicotinamide phosphoribosyltransferase **B**. Live imaging of episomally expressed GFP tagged NAD^+^ metabolic enzymes are shown (GFP-fusion proteins are shown in green). Enzyme Commission numbers are provided for each enzyme. All images are of trophozoite stage parasites. Hoechst dye (shown in blue) was used to visualize the parasite nucleus. *For example: sirtuins and poly(ADP-ribose) polymerases.

NAD^+^ can be synthesized in most organisms through both salvage and *de novo* pathways. In the human red blood cell, NAD^+^ synthesis is limited to a NAD^+^ salvage pathway that utilizes either exogenously acquired nicotinic acid (Na) or nicotinamide (Nam), which are collectively known as niacin or vitamin B_3_ [19]. Na is converted to NAD^+^ through the Preiss-Handler pathway in three steps - Na is first converted into nicotinate mononucleotide (NaMN) via the nicotinic acid phosphoribosyltransferase (NAPRT), then to nicotinate adenine dinucleotide (NaAD) via the nicotinamide mononucleotide adenylyltransferase (NMNAT) and finally to NAD^+^ via the glutamine-dependent NAD^+^ synthetase (NADSYN) [20], [21] - while Nam can be converted to NAD^+^ in a two-step pathway found in higher eukaryotic organisms involving nicotinamide riboside kinase (NRK) and NMNAT (Figure 1A) [22]. In the *de novo* synthesis pathway, prokaryotes can utilize aspartate to feed into the synthesis of NAD^+^, whereas eukaryotes rely on intermediates generated from the breakdown of tryptophan [23]. Both pathways yield NaMN, which ultimately enters into the final two steps of the salvage pathway. The *P. falciparum* genome is currently predicted to only encode the enzymes necessary for a functional NAD^+^ salvage pathway (NAPRT: PFF1410c, NMNAT: PF13_0159 and NADSYN: PFI1310w) (Figure 1A) and does not appear to possess the enzymes for *de novo* synthesis from either aspartate or tryptophan [13], [24]. While both the host and parasite possess the NAD^+^ salvage pathway, there is a significant divergence between the two pathways due to the presence of a nicotinamidase enzyme (PFC0910w) in the *P. falciparum* genome that is able to convert Nam to Na (Figure 1A).

Our current study explores the broader significance of NAD^+^ metabolism in *P. falciparum* throughout blood-stage development as a target for antimalarial intervention. Using a combination of protein biochemistry, genetics, and mass spectrometry-based metabolomics approaches, we have characterized the NAD^+^ metabolic pathway of the parasite confirming the absence of a *de novo* synthetic route. All four enzymes of the parasite salvage pathway have been localized, revealing a cytoplasmic localization for all enzymes but nicotinamidase, which localizes to the nucleus. We enzymatically characterized the parasite nicotinate mononucleotide adenylyltransferase (PfNMNAT) *in vitro* using purified recombinant protein and we demonstrate its ability to complement the *Escherichia coli* homolog *in vivo*. Following up on previous predictions that PfNMNAT is essential to *P. falciparum* development [25], we synthesized and screened derivatives of previously identified inhibitors of bacterial NMNATs [26] against purified PfNMNAT and live parasite culture. This work validates the parasite NAD^+^ metabolic pathway as a novel drug target and identifies viable lead compounds for further development as antimalarial drugs.

## Results

### Characterization of parasite NAD^+^ metabolism

Genomic reconstruction of *Plasmodium falciparum* has identified a number of enzymes involved in NAD^+^ metabolism [24], suggesting that the parasite uses a canonical Preiss-Handler salvage pathway, but lacks the enzymes required for *de novo* synthesis (Figure 1A). The parasite genome encodes for a nicotinamidase (PFC0910w, EC 3.5.1.19), nicotinate phosphoribosyltransferase (PFF1410c, EC 2.4.2.11), nicotinate mononucleotide adenylyltransferase (PF13_0159, EC 2.7.7.18), and NAD^+^ synthase (PFI1310w, EC 6.3.5.1). To identify the subcellular localization of NAD^+^ metabolism, *P. falciparum* 3D7 strains were generated expressing full-length GFP-tagged episomal copies of each enzyme and then analyzed by live fluorescence microscopy. We found that the NAD^+^ pathway enzymes are primarily localized in the cytoplasm except for the parasite nicotinamidase, which localizes to the cytoplasm but concentrates mainly in the nucleus (Figure 1B and Figure S1 in File S1).

**Figure 2:**
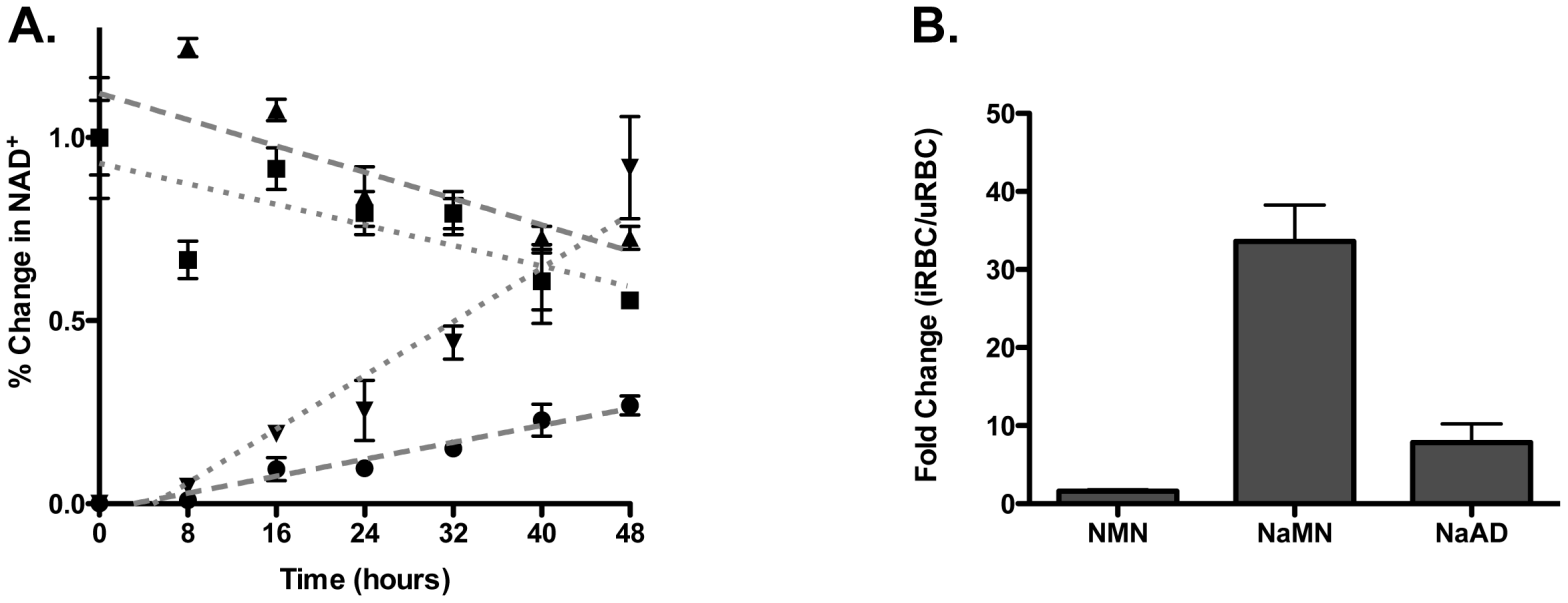
Characterization of *P. falciparum* NAD^+^ metabolism. **A**. Time course of observed percent change in NAD^+^ levels in infected and uninfected red blood cells. (▴) uRBC – NAD^+^ unlabeled, (▪) iRBC - NAD^+^ unlabeled, (▾) iRBC – NAD^+^ C^13^ labeled, (•) uRBC - NAD^+^ C^13^ labeled. Each data point is normalized to the total pool of unlabeled and labeled NAD^+^ present at time zero for the uRBC or iRBC. Error is reported as the standard deviation of three independent biological replicates. **B**. Fold Increase in Labeled NAD^+^ Intermediates in iRBCs. The ratio represents the observed level of ^13^C-labeled intermediate observed in iRBC compared to that in uRBC after a 40 hour incubation with ^13^C-U-glucose. Error is reported as the SD of three independent biological replicates.

In order to confirm the predicted architecture of the *P. falciparum* NAD^+^ salvage pathway, we grew infected red blood cells (iRBCs) in media supplemented with uniformly labeled ^13^C-U-glucose to perform stable isotopic labeling studies coupled with liquid chromatography-tandem mass spectrometry (LC-MS/MS) detection. Under these conditions ^13^C-U-labeled glucose is converted into ^13^C-U-labeled phosphoribosyl pyrophosphate (PRPP) via the pentose phosphate pathway, which is then incorporated into the nucleotide components of NAD^+^; nicotinate/nicotinamide mononucleotide (N(a)MN) and adenosine (as shown in Figure S2 in File S1). Using this approach, we are able to monitor all of the NAD^+^ salvage pathway intermediates and observe the labeling of NAD^+^ throughout the stages of the intraerythrocytic developmental cycle (IDC). The incorporation of 5+ and 10+ label into newly synthesized NAD^+^ occurs linearly in both infected red blood cells (iRBCs) and uninfected red blood cells (uRBCs), but is faster in the infected cell (d[% ^13^C-NAD+]/dt = 0.02±0.00) than the uninfected cell (d[% ^13^C-NAD+]/dt = 0.01±0.01) (Figure 2A). In addition to the steady synthesis of NAD^+^, turnover of unlabeled NAD^+^ was also observed over the 48 hour time course in both uninfected and infected cells by the steady decrease in pool of unlabeled NAD^+^ (Figure 2A). The predominant form of NAD^+^ generated in iRBCs contains newly synthesized nicotinic acid mononucleotide (NaMN) and unlabeled ATP (shown in Figure S3 in File S1). This is expected, as all NaMN made by the parasite will be incorporated into NAD^+^, while ATP is utilized in a number of additional cellular pathways.

A comparison of steady state levels of metabolic intermediates demonstrates that uninfected red blood cells preferentially generate NAD^+^ via nicotinamide mononucleotide (NMN), while the parasite produces NAD^+^ via the canonical Preiss-Handler two-step process, yielding both the labeled intermediates NaMN and nicotinate adenine dinucleotide (NaAD). This architecture was confirmed by the large fold increases observed in labeled NaMN and NaAD produced by the parasite compared to uRBCs, while labeled NMNs levels generated by the red blood cell were relatively unchanged in the presence of the parasite (Figure 2B). Therefore, the large increase in NAD^+^ concentration previously observed during Plasmodium infection is due to parasite-derived metabolism. The production of ^13^C-labeled NAD^+^ remains steady throughout the IDC in iRBCs with a total intracellular concentration of 880±100 µM during the schizont stage, almost twenty times higher than the concentration measured in uninfected controls (44±6 µM) (Table 1). While NAD^+^ levels are greatly increased in the infected red blood cell, NADP^+^ concentrations are only slightly increased from 35±1 µM to 62±12 µM upon infection (Table 1). Despite the amount of labeled NAD^+^ increasing by almost 100% in the iRBC during development (Figure 2A), only a small fraction of this pool is phosphorylated to generate labeled NADP^+^, with only 7% containing any labeled ribose (Table 1).

**Table 1:** Quantification of NAD^+^ and NADP^+^ levels in iRBCs.

	concentration (uM)	
	iRBC	uRBC
**NAD^+^**	880±100	44±6
**NADP^+^**	62±12	35±1

	% of total pool	
	iRBC	uRBC
**NADP^+^-unlabeled**	93±7	97±0.7
**NADP^+^-5 ^13^C labeled**	3.5±2.4	2.1±0.7
**NADP^+^-10 ^13^C labeled**	3.5±5	0±0

To test whether the parasite could utilize both nicotinamide and nicotinic acid to drive NAD^+^ synthesis, we performed media supplementation studies where each vitamin was provided at a standard RPMI concentration of 1 mg/mL or removed from the media to create a niacin free treatment. We observe that the parasite is able to take up and utilize both nicotinamide and nicotinic acid for NAD^+^ synthesis, with NAD^+^ levels increasing at the same rate for both forms of vitamin B_3_ (nicotinamide: d[NAD^+^]/dt = 0.09±0.01, nicotinic acid: d[NAD^+^]/dt = 0.09±0.01) (Figure S4A in File S1). When niacin is removed from the media, NAD^+^ levels remain low throughout development (Figure S4 in File S1). From this result we can confirm the lack of *de novo* NAD^+^ synthesis, as it demonstrates that the production of NAD^+^ is dependent on the availability of exogenous niacin. Therefore, during asexual development parasites are reliant on niacin uptake and can utilize both nicotinamide and nicotinic acid present in human serum to drive NAD^+^ production via the Preiss-Handler pathway.

### Enzymatic activity of PfNMNAT

**Figure 3:**
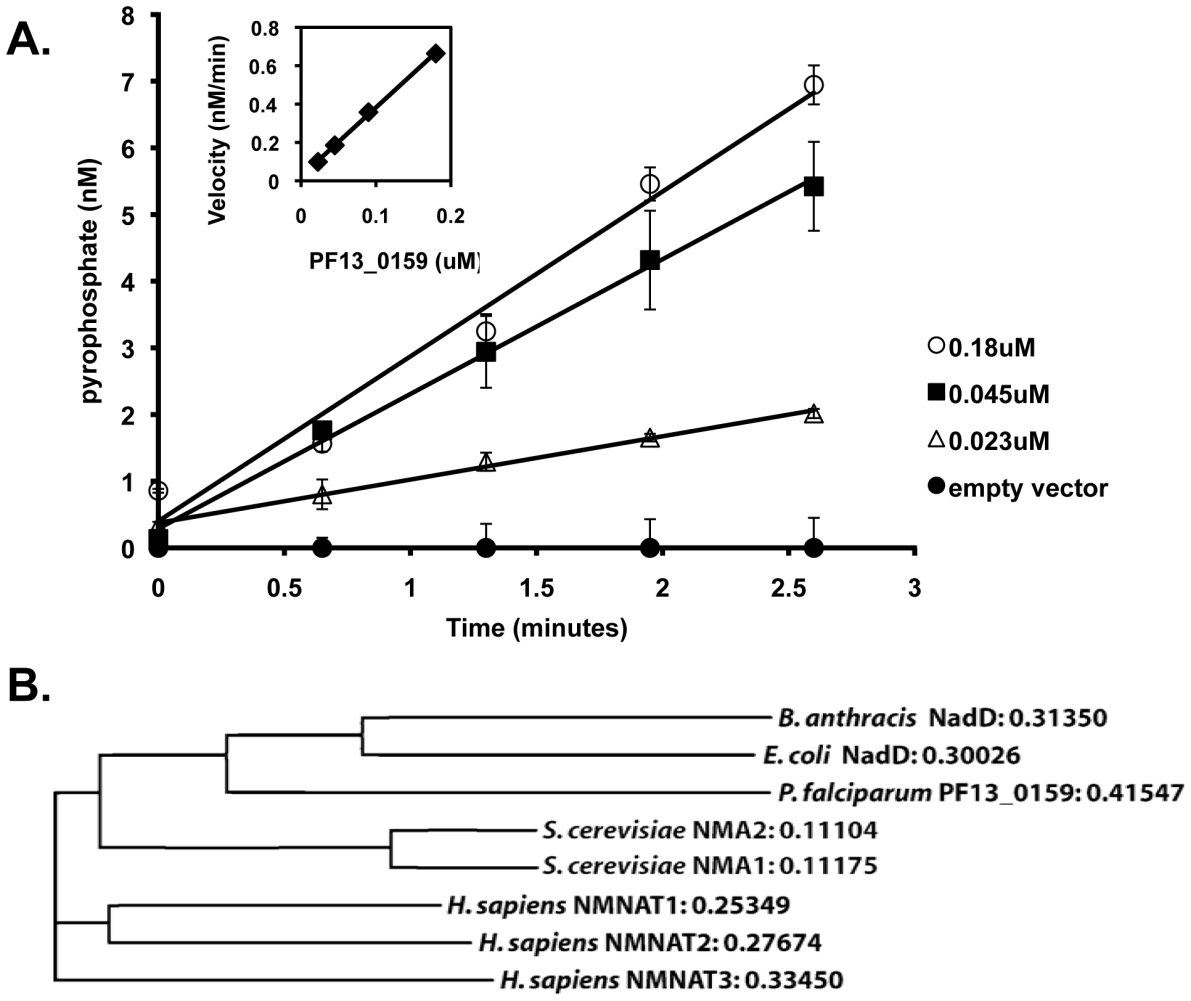
PF13_0159 encodes a nicotinate mononucleotide adenylyltransferase. **A**. Dependence of PF13_0159 adenylyltransferase activity on time and enzyme concentration. A discontinuous assay was established to measure enzyme activity; pyrophosphate release is measured as the end product of the adenylyltransferase reaction. Values were normalized to background absorbance values obtained in a buffer only control. A standard curve was generated to determine pyrophosphate concentrations. Error is reported at the SD of three independent technical replicates. **B**. Phylogenic analysis of PF13_0159 compared to representative prokaryotic and eukaryotic NMNATs. ClustalW2 was used to generate alignments and distance values are reported. An alignment of PF13_0159 and the *E. coli* NADD is shown in Figure S5 (in File S1).

To demonstrate that the predicted PfNMNAT (PF13_0159) was functional we established a discontinuous *in vitro* assay to measure the adenylyltransferase activity using purified recombinant His6×-tagged protein (Figure 3A). PfNMNAT was found to have a K_m_ of 19.9±6.7 µM for ATP and 35.8±5.1 µM for NaMN, which is comparable to the K_m_ constants reported for *Bacillus anthracis* (44 µM for ATP and 25 µM for NaMN) and other bacterial versions of this enzyme [27], [28]. We also generated an active site mutant of PfNMNAT by replacing the conserved catalytic site residue Asp-110 with an alanine. This residue is thought to either hydrogen bond with the adenosine monophosphate during the adenylyltransferase reaction or help with essential Mg^+2^ coordination during catalysis [29], [30]. The D110A mutant lacked *in vitro* activity in the enzyme assay confirming the essential function of this residue (data not shown).

### PfNMNAT complements NadD function in *Escherichia coli*

Phylogenic analysis of PfNMNAT compared to representative prokaryotic and eukaryotic NMNATs demonstrates its similarity to bacterial versions of the enzyme (Figure 3B) [31]. Based on its high level of shared identity with the *E. coli* NMNAT (NadD) (Figure S5 in File S1), we tested whether PfNMNAT could genetically complement the essential bacterial enzyme *in vivo* [32]. We generated an *E. coli* strain where the *nadD* locus was replaced with the chloramphenicol (cam) drug resistance cassette (*nadD*::cam) and the non-essential linked gene, *ybeT*, was replaced with a kanamycin (kan) drug resistance cassette (*ybeT*::kan). P1 transduction was used to move *nadD*::cam into acceptor strains carrying episomal copies of different NMNATs to test whether they could complement the deleted *nadD*. We selected transductants for the *ybeT*::kan marker and then subsequently screened for the non-selected *nadD*::cam marker. Since *ybeT* and *nadD* are linked, cam resistance should be observed in roughly 72% of the kan-resistant transductants when NadD function is supplied *in trans*; this is demonstrated in the positive control, in which 72% of kan-resistant transductants exhibit cam resistance when *E. coli nadD* is provided episomally in the acceptor strain (Table 2). Using this approach we demonstrated that PfNMNAT is able to complement NadD, as 69% of transductants had cam resistance (Table 2). Additionally, as expected, the catalytically dead D110A version of PfNMNAT was unable to complement NadD function and linkage disruption was observed. Although five cam resistant transductants were obtained, sequencing subsequently confirmed that they contained suppressor mutations.

**Table 2:** Complementation of *E. coli* NadD with PfNMNAT.

donor	acceptor (episomal copy)	selected marker	unselected marker
		*kan*	*cam*
*ybeT::kan*	*E. coli* NadD	100/100	72/100
*nadD::cam*	PfNMNAT wt	100/100	69/100
	PfNMNAT D110A	100/100	5/100
	empty vector	100/100	0/100

In order to validate our result, we tested whether PfNMNAT could rescue the *nadD* deletion strain of *E. coli* in a continuous growth assay. We placed an episomal copy of PfNMNAT into the *nadD* deletion strain under the control of the arabinose promoter, which is induced in the presence of arabinose and repressed in the presence of the competitive inhibitor fucose [33]. This strain was initially grown in the presence of arabinose and then back diluted into LB containing arabinose, fucose, or no inducer. When grown in the presence of the inducer arabinose, PfNMNAT is able to rescue growth more efficiently than in the no-inducer control (Figure S6 in File S1). When PfNMNAT expression is suppressed in presence of fucose, growth is not rescued and lags behind the control condition (Figure S6 in File S1). Taken together, both experimental approaches demonstrate the ability of PfNMNAT to complement *E. coli* NadD. This demonstrates functional conservation between the *P. falciparum* and *E. coli* enzymes and suggests that much of what is known about the well-characterized bacterial homologs of this enzyme may apply to PfNMNAT.

### NMNAT inhibitors disrupt *Plasmodium falciparum* growth in vivo

**Figure 4:**
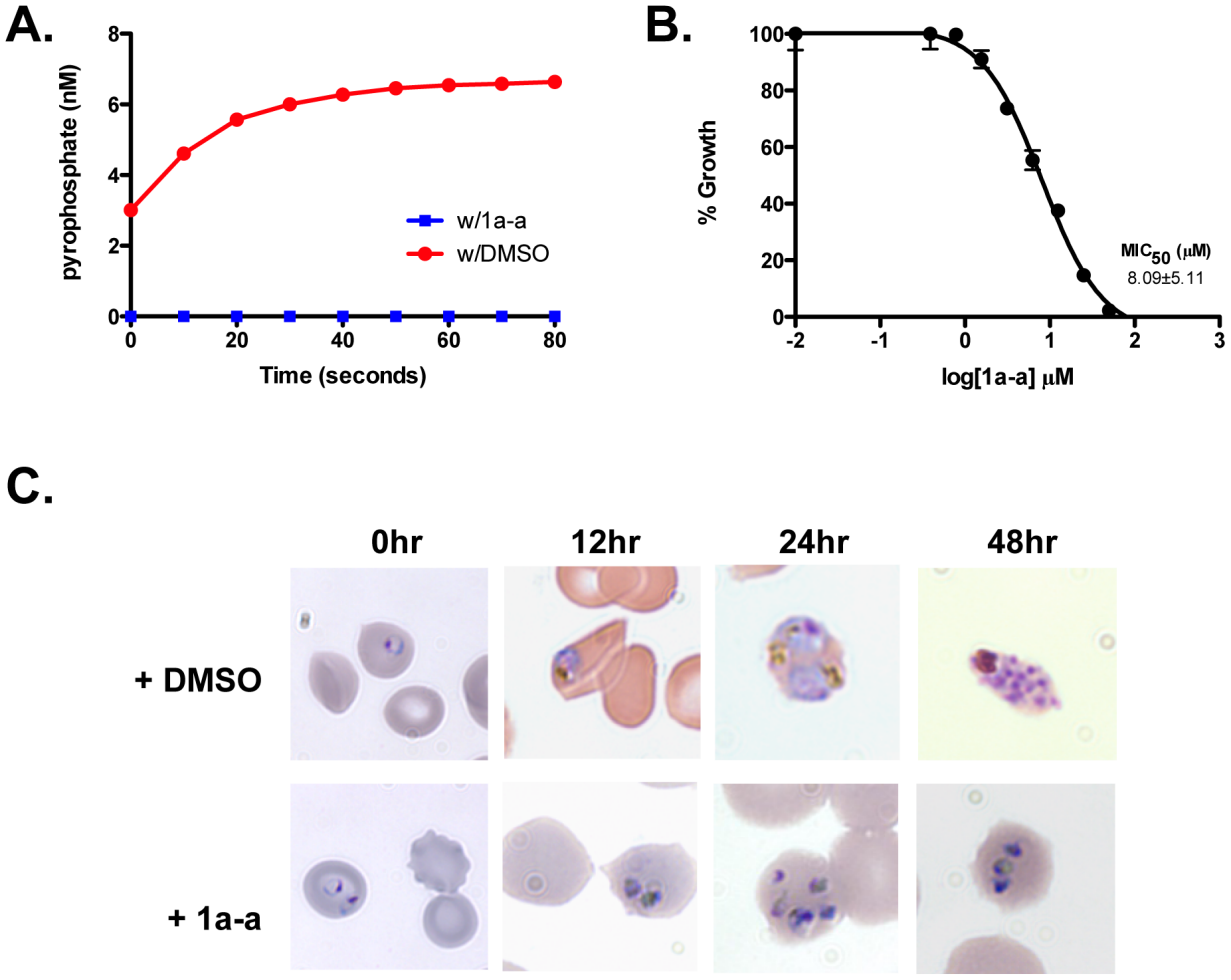
Inhibitory effect of putative NMNAT inhibitors *in vitro*. **A**. 1a-a inhibits adenylyltransferase activity of PfNMNAT. Purified enzyme was preincubated with either 50 µM 1a-a or 2% of DMSO for ten minutes before pyrophosphate release was monitored continuously at 565 nm. A standard curve of pyrophosphate was used to determine concentration from observed absorbance values. Error is reported at the SD of three independent technical replicates. **B**. MIC_50_ curve for 1a-a. A standard SYBR green growth assay was performed on synchronous ring stage parasites to determine MIC_50_ (See Material and Methods). Error is reported as the SD of three independent biological replicates. **C**. Imaging of *P. falciparum* treated with 50 µM of 1a-a or a 1% DMSO control. Parasite growth was monitored by imaging of fixed parasites visualized with Giemsa stain.

Based on a previous report that demonstrated the anti-parasitic activity of the known bacterial NMNAT inhibitor, compound 1_03 [25], renamed 1a-a in this study, we sought to characterize the specificity of this compound further. Using our established enzymatic assay, we found that 1a-a exhibits specific activity against purified his-PfNMNAT (Figure 4A), and inhibits parasite growth *in vivo* with an MIC_50_ of 8.09±5.11 µM (Figure 4B). When 1a-a is added to synchronous ring stage cultures at 50 µM, parasite growth arrests early in development (Figure 4C).

**Figure 5:**
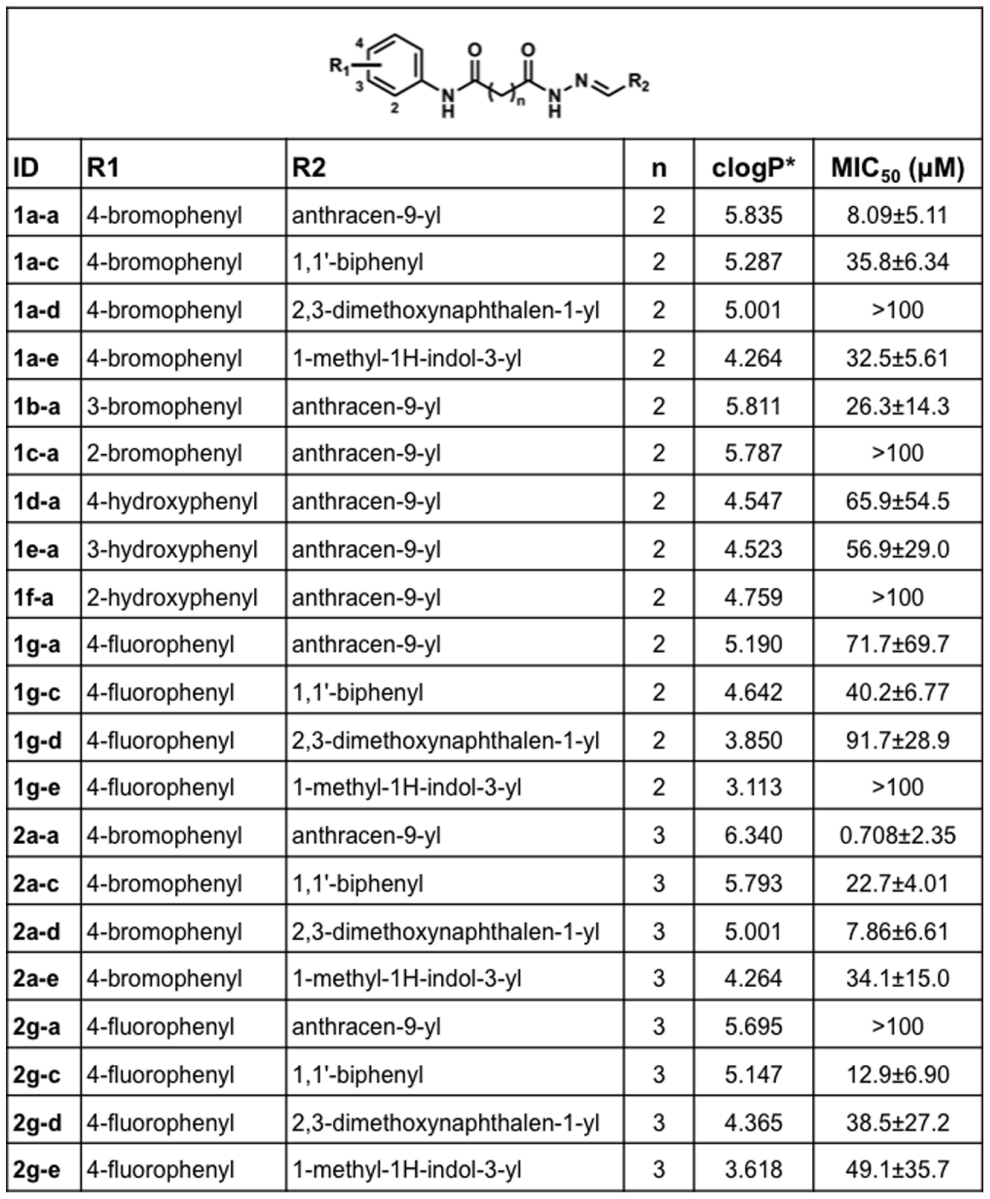
Antimalarial activity and partition coefficients for putative NMNAT inhibitors. MIC_50_ values were determined for each synthesized NMNAT inhibitor using a standard SYBR green growth assay on synchronous ring stage parasites. Error is reported as the SD of three independent biological replicates. *Calculated octanol/water partition coefficients (cLogPs) for all compounds were determined using online tools at http://www.molinspiration.com.

In order to attempt to improve certain features of the 1a-a scaffold design, we first developed an efficient chemical synthesis pathway to produce 1a-a. With a streamlined synthetic scheme in place, we utilized the chemical backbone of 1a-a (shown in Figure 5) to generate a number of derivatives to screen against live *P. falciparum* culture. Our approach was to improve the observed MIC_50_ of 1a-a derivatives by enhancing both their solubility and binding affinity. Solubility improvement was attempted principally by replacing hydrophobic groups with more hydrophilic moieties. Binding affinity was approached by estimating the points of likely enzyme-inhibitor interaction based on the co-crystal of the inhibitor bound to the bacterial NMNAT (PDB code MLA3) [34] and attempting to alter the spatial positioning at these points. To achieve these aims three regions of the molecular scaffold were modified and tested, the aniline moiety (R_1_), the aromatic imine (R_2_) and the central carbon chain (n), resulting in over twenty novel compounds (Figure 5). All of the compounds were solubilized in dimethylsulfoxide (DMSO) and tested against live, synchronous *P. falciparum* 3D7 culture to determine experimental MIC_50_ concentrations, which are reported in Figure 5.

We chose to modify the aniline group (R_1_) because differentially substituted anilines have previously been tested against bacterial NMNAT and have yielded strong inhibition (Sorci et al., 2009). The MIC_50_ values obtained for these substitutions (Figure 5) did not improve upon the lowest MIC_50_ of 8.09±5.11 µM observed for the 4-bromoaniline substitution, therefore this R_1_ group was utilized in further compound development. We then tested a number of aromatic aldehydes at the R_2_ position, all imparting greater compound solubility than the anthracen-9-yl. These compounds were all effective in inhibiting parasite growth with the lowest MIC_50_ of 7.86 µM observed for 2a-d (Figure 5).

The original 1a-a compound was determined to have a calculated logP (clogP) solubility index value of 5.835 (Figure 5). Hydrophilic residues were incorporated into derivative compounds in an attempt to increase solubility (as reflected in lower clogP values) to obtain lower MIC_50_ values. The lowest clogP value of 3.113 was obtained for compound 1g-e (Figure 5), with the highest clogP value of 6.34 corresponding to the 2a-a. The trend of decreasing the clogP values in the derivative compounds resulted in higher MIC_50_ values, suggesting that these compounds were less effective in binding PfNMNAT.

We also made one modification to the carbon linker between the R_1_ and R_2_ groups with the addition of an extra carbon unit, with the prediction that this extension would allow for a better conformational fit in the pocket between the two active sites. Remarkably, the 2a-a compound differed from 1a-a only by the added carbon unit gave the greatest improvement with a MIC_50_ value of 0.708±2.35 µM) (Figure 5). Overall, six of the eight compounds where the carbon linker was modified had lower MIC_50_ values when the extra carbon unit was present (Figure 5). In summary, twenty different compounds were synthesized representing a wide range of functional group substitutions and clogP values.

## Discussion

In this study, we utilized various experimental approaches to characterize the NAD^+^ biosynthetic pathway of *Plasmodium falciparum*. Bioinformatic analysis identified the components of a canonical salvage pathway in the parasite, but did not identify the enzymes involved in *de novo* synthesis, suggesting that the parasite is a NAD^+^ auxotroph [24]. To confirm the architecture of the parasite's NAD^+^ synthetic pathway, stable isotope labeling via fully labeled ^13^C-U-glucose was used to trace labeled carbon into newly synthesized NAD^+^ throughout development. This demonstrated the steady and continuous synthesis of NAD^+^ throughout the 48-hour parasite life cycle as well as showing a steady rate of turnover of unlabeled NAD^+^. Both the rate of synthesis and total concentration of NAD^+^ are much higher in infected red blood cells when compared to uninfected control cultures. Coupled with the observation of increased levels of labeled NaMN and NaAD in iRBCs, we can conclude that the high levels of NAD^+^ are being synthesized and utilized by the parasite and not the host cell during infection. This is finding is supported by previous reports of elevated level of NAD^+^ in infected erythrocytes and the observation that nicotinamidase activity, an enzyme found in the parasite and not in the host, is increased in *P. falciparum* infected red blood cells [9]. The measured intracellular NAD^+^ concentrations are also in agreement with those reported in a NMR spectroscopy study of purified parasites and uninfected RBCs, which also found higher NAD^+^ levels in isolated trophozoites [35]. Elevated NAD^+^ synthesis driven by the parasite suggests that the NAD^+^ pathway can be pursued as a viable therapeutic target without compromising host erythrocyte metabolism.

Despite a ten-fold difference in NAD^+^ levels, we observe little difference in the concentration of NADP^+^ between iRBCs and uRBCs. Even more striking is the small percentage of the newly synthesized NAD^+^ that becomes phosphorylated. Although a putative NAD^+^ kinase (PFI0650c, EC 2.7.1.23) and pyridine nucleotide (NADP^+^) transhydrogenase (PF14_0508, EC 1.6.1.2) have been identified, they have not been characterized. NADPH is utilized by both the glutathione and thioredoxin systems in the parasite to deal with oxidative stress [36]. It is additionally utilized in a number of anabolic pathways, such as isoprenoid and fatty acid synthesis [37], [38]. In all of these processes, NADP^+^ is cycled between its oxidized and reduced states without being consumed. It is therefore possible that the amount of NADP(H), while much less than the total pool of NAD^+^, is sufficient for blood stage development.

Gene expression data [39], [40] for the NAD^+^ metabolic enzymes have suggested that they have unusual and uncoordinated bi- and mono-phasic transcriptional profiles. The parasite nicotinamidase and NMNAT exhibit transcriptional peaks around 20 and 40 hours post invasion, while the nicotinate phosphoribosyltransferase and NAD^+^ synthase enzymes are maximally transcribed between 25–28 hours. Although it has been proposed [41] that NAD^+^ synthesis would peak with NAD^+^ synthase expression, we observe steady NAD^+^ production throughout the IDC. This is likely necessary to maintain NAD^+^ pools to replace the NAD^+^ that is lost to turnover and provides a sufficient increase in total NAD^+^ required for the development of new merozoites.

We were able to confirm the parasite's reliance on uptake of exogenous niacin in order to synthesize NAD^+^ via the canonical Preiss-Handler salvage pathway. When niacin was removed from the culture media, intracellular concentrations of NAD^+^ were significantly lower. However, in agreement with previous nutrient limitation studies, niacin deprivation did not lead to a growth defect *in vitro* suggesting that the niacin provided by the host cell is sufficient to provide the required amount of NAD^+^ synthesis necessary for growth *in vitro* [42]. This is unsurprising during the asexual and sexual stages in the human host, where the parasite is developing in the niacin rich environments of human hepatocytes, red blood cells and serum [43]. Furthermore, a reliance on niacin salvage has been shown for other pathogenic organisms, such as *Leishmania infantum* and multiple species of pathogenic yeast, making it a common evolutionary adaptation [44], [45]. In multiple species of yeast, niacin transporters have been characterized and shown to be upregulated in niacin starvation conditions [46], [47]. To date, however, no homologs of these identified transporters have been identified in the *Plasmodium* genome. The parasite's reliance on a salvage pathway and dependence on the availability of niacin makes it increasingly susceptible to multiple points of interventions.

NMNATs have emerged as an attractive drug target due to their indispensible role in NAD^+^ synthesis, catalyzing the essential step shared by both the *de novo* and salvage pathways [48], [49]. Our biochemical characterization of PfNMNAT validates its function as a *bona fide* nicotinate mononucleotide adenylyltransferase and demonstrates the essentiality of the conserved active site Asp110 residue for its catalytic activity. Proteomic data has detected the presence of PfNMNAT peptides during both the asexual and sexual stages further supporting its role in these life stages and suggesting that potential inhibitors could target both important life stages [50], [51]. Furthermore, PfNMNAT is well conserved across all species of *Plasmodium* as well as in other parasitic organisms in the Apicomplexan phylum such as *Babesia bovis* and *Theileria annulata*, [52], [53] implicating NMNATs as a drug target beyond *P. falciparum*.

In this study, we demonstrated the high level of conservation between the parasite NMNAT and its bacterial homolog by complementing the essential *E. coli* NadD *in vivo*. This similarity is striking when compared to the high level of divergence between the parasite and the three human NMNATs as demonstrated by an alignment of the four protein sequences (Figure S7 in File S1). Previous comparison of the solved *E. coli* and human NMNATs crystal structures has shown significant structural divergence between their respective active sites, suggesting the ability to selectively target the bacterial enzymes [26]. Due to both the similarity of the parasite PfNMNAT to its bacterial homologs and its divergence from the host enzyme, it presents a new target for chemical inhibition. Inhibitors of bacterial NMNATs have been previously identified based on *in silico* screening of databases of compounds against solved three-dimensional protein structures. These compounds were then further tested both *in vitro* and *in vivo* to identify lead compounds for drug development and select for those that demonstrated no inhibitory effect on the human NMNATs [26], [34]. A structure of a co-crystal of the bacterial NMNAT and one of the most effective inhibitors, compound 1a-a, suggested that this compound and those closely related to it, bind to the enzyme in its *apo* form and interact with both the NaMN binding pocket and the ATP binding site [34]. Since both regions are conserved in PfNMNAT we reasoned that such inhibitors might interact with the parasite enzyme in a similar fashion and prove effective in inhibiting growth *in vivo*, without impacting host metabolism.

Starting with the chemical backbone of the original 1a-a compound, we made a number of modifications to generate the derivatives tested in this study. We attempted to increase compound solubility, as reflected in lower clogP values, in order to increase drug potency. We synthesized compounds in a range of clogP values from 6.34–3.113 (Figure 5), which resulted in the opposite effect, with the highest clogP values corresponding to the lowest MIC_50_ values. This could be the result of a number of different factors such as a hydrophobic effect on binding, disruption of important hydrogen bonds within the active site and other steric effects.

Multiple modifications were made to the R_1_ group. In Huang et al., a solved structure with 1a-a and the *B. anthracis* NMNAT demonstrated an interaction between the para positioned bromine and the His18 residue in the T/HXGH motif. None of our R_1_ modifications resulted in significant improvement over the MIC_50_ value of 1a-a, suggesting that the para positioned bromine allows for the best interaction with the ATP binding site. The polyaromatic moiety present as the R_2_ group in 1a-a was shown by crystallography to exhibit π-stacking with up to three aromatic residues of the *B. anthracis* NMNAT [34] in the nicotinosyl binding pocket. To optimize this effect, a number of aromatic aldehydes were tested at the R_2_ position. These moieties imparted greater compound solubility as reflected by their lower calculated partition coefficient (clogP) values, but were not found to significantly lower MIC_50_ values (Figure 5). The addition of an extra carbon unit to the linker between the R_1_ and R_2_ groups was predicted to allow for a better conformational fit in the pocket between the two active sites. This single modification is the sole difference between the 2a-a and 1a-a compounds and resulted in almost a ten-fold improvement in its MIC_50_ value (Figure 5). Overall, this modification presented the best improvement to potency obtained for the compounds generated and tested in this limited study, even though many of the synthesized compounds exhibited modest antimalarial activity. Without a solved crystal structure of PfNMNAT it is hard to correlate MIC_50_ with the specific features of compound structure, especially the novel functional groups utilized in our study. Moving forward it will be essential to further characterize both the PfNMNAT enzyme and explore further chemical modifications to this identified compound scaffold in order to generate viable compounds for drug development. Additionally, any identified lead compounds will need to be screened for potential activity against the human NMNATs to ensure they do not display cross-reactivity.

In summary, this work highlights NAD^+^ metabolism as a new area of interest in *Plasmodium* biology and potentially other closely related Apicomplexan organisms. The identification of both novel drug targets and putative lead compounds is significant at a time when drug resistance is on the rise and few lead antimalarial compounds are in later stages of drug development.

## Materials and Methods

### *P. falciparum* Culture

The *P. falciparum* 3D7 and D10 clones were cultured and synchronized by standard methods [54], [55] with the modification that culture flasks were maintained at 37° in an atmospherically controlled incubator set at 5% CO_2_, 6% O_2_. Synchronicity and parasitemia was monitored using microscopy and Giemsa stained smears. *P. falciparum* strains used in this study were obtained from the Malaria Research and Reference Reagent Resource Center (MR4, www.mr4.org), which is a part of the NIAID BEI Resources collection.

For niacin deprivation experiments standard RPMI media [56] was reconstituted using 50× RPMI Amino Acid Solution (Sigma Aldrich) and individual vitamins and salts purchased from Sigma Aldrich and Fisher Chemical. Fully reconstituted RPMI was always made in parallel and used as a control to ensure there was no variation in parasite growth between batches.

### Cellular localization of GFP-tagged NAD^+^ metabolism enzymes

The four full length gene loci corresponding to the enzymes of the *Plasmodium* NAD^+^ salvage pathway (*pf13_0159*, *pff1410c*, *pfi1310w*, *pfc0910c*) were cloned into the pDC2-CAM-CRT-GFP plasmid [57] to create C-terminal GFP fusions under the control of the *P. falciparum* calmodulin promoter (*pf14_0323*) using the F and R pDC2 (Table S1 in File S1). These plasmids were transfected via electroporation into ring stage D10 culture with a BioRad GenePulser and maintained episomally under WR99210 selection [58]. Live parasites were stained with Hoechst dye to visualize the nucleus and then imaged with a Leica microscope equipped with SlideBook software to determine the localization of the protein-GFP fusions. Protein-GFP fusions were also validated by Western blot. Lysates were prepared from mixed stage culture and separated on 10% denaturing Tris-glycine gels. Lysates were transferred to nitrocellulose membrane using the wet transfer method overnight at 4°, 20 V and probed with Roche anti-GFP antibody (11 814 460 001) at 1∶1500, 1 h, followed by probing with Sigma anti-rat IgG (whole molecule) peroxidase conjugate (A 9037) at 1∶2000, 45 min. The signal was visualized using Pierce ECL reagent.

### LC-MS/MS detection of NAD^+^ intermediates

The *P. falciparum* 3D7 clone was cultured under standard conditions at a starting parasitemia of 6% and hematocrit of 2%. Isotopic labeling was initiated by resuspending cultures in RPMI containing ^13^C-U-glucose. A parallel uninfected erythrocyte culture (from the same blood donor) was treated identically and samples were collected every 8 hours from both iRBC and uRBC cultures. Metabolomic extraction, LC-MS/MS instrumentation, and data extraction was performed as described previously [7]. Briefly, cultures were centrifuged and the cell pellet was extracted first into 4 volumes of 100% methanol on dry ice and then with one volume of ice-cold 80∶20 methanol∶water in a water bath sonicator. Samples were dried down under nitrogen and resuspended in water prior to analysis. LC-MS/MS was performed using a Shimadzu LC-10A HPLC system and a Phe-nomenex Luna aminopropyl column (250 mm 3×2 mm with a 5 mm particle size) coupled to a Finnigan TSQ Quantum Ultra triple quadrupole mass spectrometer (Thermo Electron Corporation) running in positive mode. Data was collected and processed by Xcalibur software (Thermo Electron Corporation). All data is representative of the standard deviation of three biological replicates.

To determine the intracellular concentration of NAD^+^ and NADP^+^ two methods were taken. Absolute quantification was performed via ^13^C-labeling for 72-hours as described in (Bennett et al., 2008). Once the intracellular metabolite pools were close to completely ^13^C-labeled, metabolites were extracted and spiked with unlabeled NAD^+^ and NADP^+^ in a dilution series. Unlabeled NAD^+^ and NADP^+^ standard curves were produced and the biological (^13^C-labeled) NAD^+^ and NADP^+^ concentrations were determined using a linear fit. Final intracellular concentrations were determined by determining the total cell number via hemocytometer cell counting and assuming the intracellular volume of an infected red blood cell is 75 fL [59]. The second approach was measuring the intracellular NAD^+^ and NADP^+^ under unlabeled conditions. Four identical samples from each iRBC and uRBC cultures were taken and subsequently spiked in with different concentrations of unlabeled NAD^+^ and NADP^+^. Total ion counts were collected in the dynamic range via positive mode LC-MS and the cellular background subtracted from the spike-in samples. A standard curve was constructed and intracellular concentration determined as described above.

### Purification of PfNMNAT recombinant protein

The *pf13_0159* open reading frame (615 bp) was cloned into pProEX (Invitrogen) in order to generate a N-terminal 6× HIS tag fusion using the *pf13_0159* pProEX F and R primers (Table S1 in File S1). Recombinant protein was expressed in *Escherichia coli* BL21 (RIL Codon PLUS) (Stratagene) cells grown at 25° and induced with 0.5 mM Isopropyl β-D-1-thiogalactopyranoside (IPTG) overnight. The induced culture was pelleted, resuspended in buffer (20 mM imidazole in 1× PBS) and then mechanically lysed. Protein was purified over Ni Sepharose 6 Fast Flow resin (GE Healthcare) and eluted with 500 mM imidazole phosphate buffer. Purity of the 26 kDa fusion protein was verified by silver stain SDS/PAGE.

To generate the PfNMNAT D110A mutation, site directed mutagenesis was used. The wild type PF13_0159 locus was subcloned into the StrataClone vector (Agilent Technologies) and PCR was performed using the overlapping primers, D110A SENSE and ANTISENSE (Table S1 in File S1), containing the desired mutation. To recover the mutated plasmid, the native template was digested with DpnI (New England Biolabs) and then transformed into *E. coli* strain DH5α.

### PfNMNAT enzymatic assays

Prior to assays, purified PfNMNAT-6×HIS protein was dialyzed into reaction buffer containing 100 mM HEPES and 10 mM MgCl_2_ at pH 7.5 overnight at 4° using 3500 Da MWCO dialysis tubing from Fisherbrand. A discontinuous assay was set up to determine the steady-state kinetic parameters for PfNMNAT as previously described [26]. This approach couples the release of the byproduct pyrophosphate to the colorimetric detection of free phosphate.

1. NaMN adenylyltransferase reaction: NaMN+ATP→NaAD^+^+PP_i_
2. Inorganic pyrophosphatase reaction: PP_i_+H_2_O→2P_i_

Pyrophosphate release was visualized using the P_i_Per Pyrophosphate Assay Kit (Invitrogen). 100 µL reactions were set up in 96-well plates (BD Biosciences) according to manufacturer specifications. Nicotinic acid mononucleotide was obtained at maximum purity from Sigma Aldrich. ATP was purchased from Fisher Bioreagents. Steady-state kinetic analysis of PfNMNAT was performed by varying each substrate concentration (NaMN and ATP) in a range from 0 to 500 µM, while providing the other substrate at 500 µM. Assays were monitored continuously at 565 nm using a BioTek Synergy MX plate reader equipped with Gen5 software. To subtract for any background activity present in the active fraction, an empty vector control was purified following the same protocol. Apparent K_m_ values were calculated by fitting the data to a standard Michaelis-Menten model using GraphPad Prism Version 5.0 software.

### Genetic complementation of the *E. coli* NadD with PfNMNAT

The *nadD* locus from *E. coli* (642 bp) was cloned into pBAD18 [33], [60] using the ecNadD pBAD F and R primers (Table S1 in File S1) to generate pNadD, which was transformed into the *E. coli* strain MC4100 containing the linked *ybeT*::cam marker (strain JO1, Table S2 in File S1). Chromosomal *nadD* was replaced by a kanamycin resistance cassette amplified using the primers CamKanNadD F and R primers (Table S1 in File S1) in the *E. coli* strain DY378 (Yu et al., 2000) containing pNadD by lambda Red recombineering [61]. The resulting *nadD*::kan knockout strain (JO2, Table S2 in File S1) was used as a donor for P1-mediated transduction [62]. Designated acceptor strains contained episomal copies of either *E. coli nadD* or PfNMNAT, and transductants were selected on LB agar containing kanamycin and appropriate inducer (L-arabinose or IPTG). Growth assays were conducted in 96 well plates and continuously monitored for absorbance at 600 nm using a BioTek Synergy MX plate reader equipped with Gen5 software for 10 hours. *E. coli* containing pBAD18::PfNMNAT were grown in the presence of arabinose to late log phase and then diluted into LB or LB containing 0.2% (g/mL) arabinose or 0.05% (g/mL) fucose.

### Synthesis of NMNAT inhibitors

All reactions were carried out under an inert atmosphere of nitrogen unless otherwise indicated. Tetrahydrofuran (THF) and dichloromethane (DCM) were purified by passage of the argon-purged solvents through activated alumina columns. Other commercial reagents were used as received, without further purification. Reactions were monitored by thin layer chromatography (TLC) performed on 0.25 mm Whatman silica gel places (60 Å) with fluorescent indicator using UV light as a visualizing agent. The plates were subsequently developed using basic potassium permanganate, ceric ammonium molybdate or *p*-anisaldehyde stain.

Nuclear magnetic resonance (NMR) spectra were obtained on 500 MHz Bruker AVANCE and Varian Unity/INOVA spectrometers. ^1^H NMR spectra are referenced to the residual monoprotio-solvent peak ((CD_3_)_2_SO: 2.50 ppm) and ^13^C NMR are referenced to the deuterated solvent signal ((CD_3_)_2_SO: 39.52 ppm). Multiple shifts arriving from conformational isomers are separated by a slash (shift 1/shift 2). Multiplicities are abbreviated as follows: s = singlet, d = doublet, t = triplet, q = quartet, m = multiplet, br = broad signal. High-resolution mass spectra were obtained on an Agilent 6210 High-Resolution Time-of-Flight mass spectrometer coupled to an Agilent Technologies 1200 series High Performance Liquid Chromatography system using no HPLC column. Details specific to the synthesis of each compound are presented in the Supporting Info.

### *P. falciparum* growth inhibition assays with PfNMNAT inhibitors

Growth inhibition assays were conducted as standard SYBR green I fluorescence assays [63] with the noted alterations. 200 µL aliquots of 0.5% synchronous ring stage culture at 2% hematocrit were set up in 96-well plates and incubated for 72 hours at standard culture conditions with varying concentrations of drug dissolved in DMSO. NMNAT inhibitors were resuspended to make 10 mM stock solutions. After growth, plates were placed at −80° overnight and thawed at room temperature. Once lysed, 100 uL of culture was incubated with SYBR green I dye (Sigma) (0.4 µL/mL) in 100 µL of buffer (20 mM Tris-HCl, pH 7.5; 5 mM EDTA; 0.08% Triton X-100; 0.008% saponin). Fluorescence was quantified (excitation: 485 nm; emission: 535 nm) using a BioTek Synergy MX plate reader equipped with Gen5 software. Assays were always run in parallel with a chloroquine treated control growth assay.

## Supporting Information

File S1 **Contains the files: Figure S1. PfNico (PFC0910w) localization throughout the IDC.** Live imaging of episomally expressed enzyme-GFP fusion proteins in the parasite. **Figure S2. Labeling pattern of NAD^+^.** The resulting labeling pattern of NAD+ from C13-U-glucose is depicted. **Figure S3. Observed labeling pattern of NAD^+^ in iRBCs.** Comparison of half and full labeled NAD+ generated in iRBCs in the presence of C13-U-glucose. **Figure S4. NAD^+^ synthesis under different niacin conditions.** Observed fold change in NAD+ concentration resulting from different niacin present in the culture medium. **Figure S5. Alignment of PfNMNAT and the** ***E. coli*** **homolog NadD.** Comparison of the amino acid sequences of the parasite and bacterial NMNAT enzymes. **Figure S6. Complementation of** ***E. coli*** **NadD with PfNMNAT.** The ability of the parasite NMNAT to rescue *E. coli* growth is demonstrated in a continuous growth assay. **Figure S7. Alignment of PfNMNAT and the Human NMNAT homologs.** Comparison of the amino acid sequences of the parasite and human NMNAT enzymes. **Table S1. Primers Used in This Study. Table S2. Strains Used in This Study.**
(PDF)
