# Supplementary material for: Targeting NAD+ Metabolism in the Human Malaria Parasite Plasmodium falciparum
Source: PLoS One. 2014 Apr 18;9(4):e94061. doi: 10.1371/journal.pone.0094061 (PMC3991606; doi:10.1371/journal.pone.0094061)
Supplement: File S1 — Contains the files: Figure S1. PfNico (PFC0910w) localization throughout the IDC. Live imaging of episomally expressed enzyme-GFP fusion proteins in the parasite. Figure S2. Labeling pattern of NAD+. The resulting labeling pattern of NAD+ from C13-U-glucose is depicted. Figure S3. Observed labeling pattern of NAD+ in iRBCs. Comparison of half and full labeled NAD+ generated in iRBCs in the presence of C13-U-glucose. Figure S4. NAD+ synthesis under different niacin conditions. Observed fold change in NAD+ concentration resulting from different niacin present in the culture medium. Figure S5. Alignment of PfNMNAT and the E. coli homolog NadD. Comparison of the amino acid sequences of the parasite and bacterial NMNAT enzymes. Figure S6. Complementation of E. coli NadD with PfNMNAT. The ability of the parasite NMNAT to rescue E. coli growth is demonstrated in a continuous growth assay. Figure S7. Alignment of PfNMNAT and the Human NMNAT homologs. Comparison of the amino acid sequences of the parasite and human NMNAT enzymes. Table S1. Primers Used in This Study. Table S2. Strains Used in This Study. (PDF) [file pone.0094061.s001.pdf]

Supporting Information

Supplemental Figures

Figure S1. PfNico (PFC0910w) localization throughout the IDC.
A. Live imaging of the episomally expressed PfNico-GFP fusion. The nucleus is visualized with Hoechst staining. **B.** α-GFP western blot verification of PfNico (50 kDa) + GFP (26 kDa) = fusion protein (76 kDa)

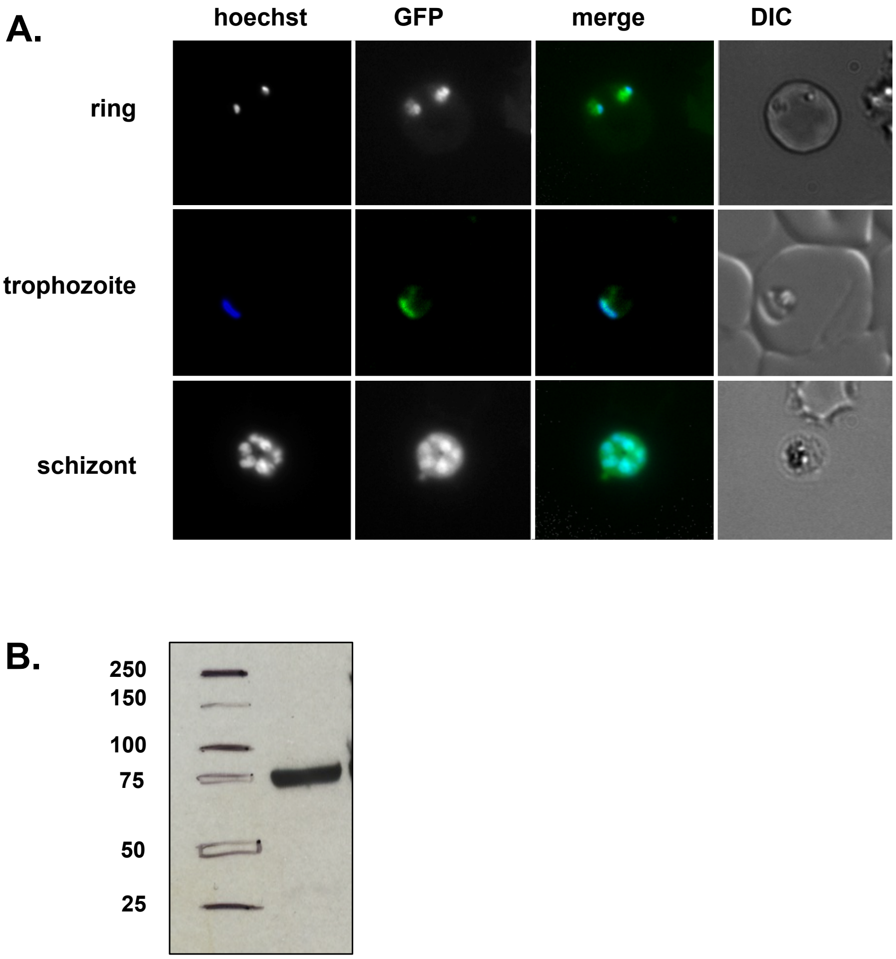

Figure S2. Labeling pattern of NAD^+.
In the presence of C13-U-glucose, full-labeled phosphoribosyl pyrophosphate (PRPP) is generated via the pentose phosphate pathway, which contributes the ribose group for both the nicotinamide mononucleotide (NMN) and adenosine portion of NAD^+. Newly synthesized NAD+ can either be labeled at one or both sugar molecules, resulting in a measurable mass increase of 5 or 10. Labeled carbons are indicated in red.

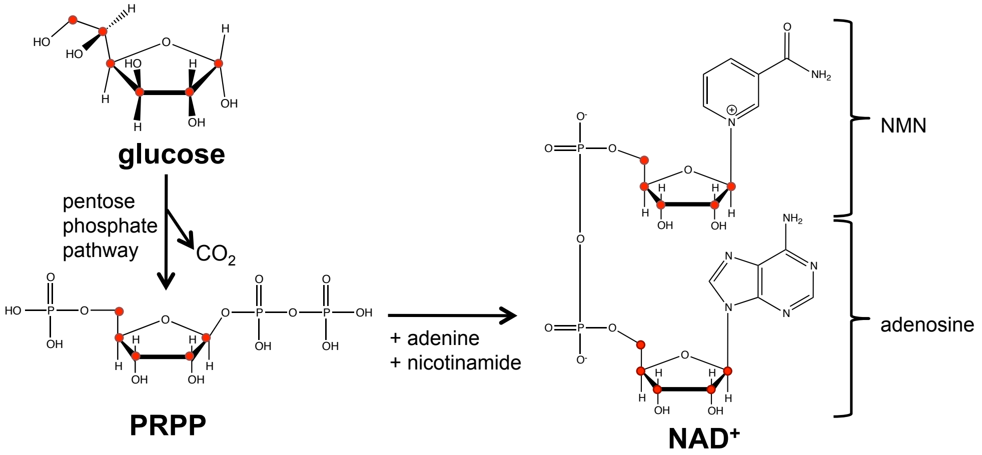

Figure S3. Observed labeling pattern of NAD^+ in iRBCs.
(●) Half NAD+ labeled at NMN ribose, (▲) Half NAD+ labeled at adenosine ribose, (■) Full-labeled NAD+ at both nucleotides. Values are normalized to the observed concentrations at time 0hr. Error is reported as the SD of three independent biological replicates.

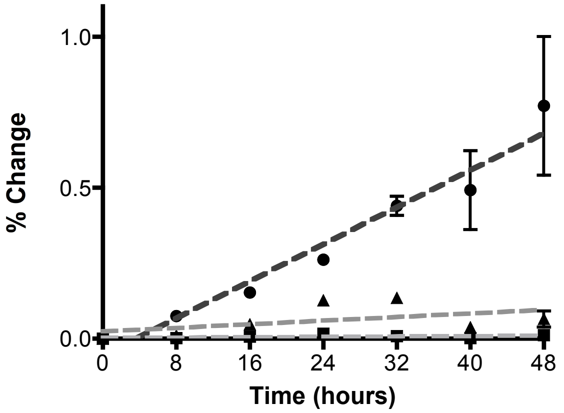

Figure S4. NAD$^+$ synthesis under different niacin conditions.
(●) RPMI + nicotinamide, (■) RPMI + nicotinic acid, (▲) RPMI without niacin. iRBC were cultured in the indicated conditions and metabolite samples were collected to determine NAD$^+$ levels at each time point. Values are normalized to the observed concentrations at time 0hr and error is provided as the SD of three independent biological replicates.

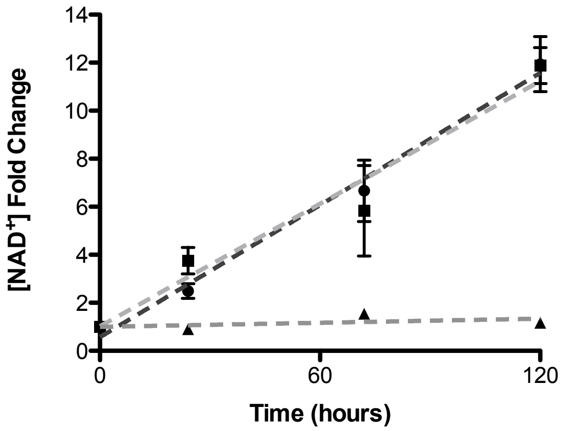

Figure S5. Alignment of PfNMNAT and the *E. coli* homolog NadD.
ClustalW2 was used to generate the alignment between the *P. falciparum* NMNAT (NCBI Gene ID: 814129) and the *E. coli* NMNAT (NCBI Gene ID: 953896). Conservation of canonical ATP binding motif ((H/T)xGH) is highlighted in red. The conserved catalytic site aspartic acid residue is highlighted in blue.

```
PfNMNAT         MHKNICIYGGSFDPITYAHEMVLDKISNLNWIHEIWVVICRCRNDKSLTEFHHRHNMFTI 60
E. coli NadD    MKSLQALFGGTFDPVHYGHLKPVETLANLIGLTRVTIIPNNVPPHRPQPEANSVQ-RKHM 59
                *:.   : **:***  *.*    : : :   : .: .:  .   .:   :    .    :

PfNMNAT         IINNSSKIIKSKIFLKDLESHSEMTPTYDLLKTQKELHPNYTFYFGLGSDLICDIFSWDE 120
E. coli NadD    LELAIADKPLF-LDERELKRNAPSYTAQTLKEWRQEQGPDVPLAFIIGQDSLLTFPTWYE 118
                :           :  .:*. :.   .   : :  ::  *:  : * :* * :  : .* :

PfNMNAT         GEKLVLENAFIIIERGHFKIDESILKKFP--------------KYYLINIPKLSFINFI 165
E. coli NadD    YETILDNAHLIVCRRPGYPLEMAPQYQQWLEDHLTHNPEDLHLQPAGKIYLAETPWFN-I 177
                 * ::    ::   *  : :  .                         *   : . :  .

PfNMNAT         SSSEARK-FLTKENDINDIKKYIHPLTIDYIIKYNLYDFN 204
E. coli NadD    SATIIRERLQNGES----CEDLLPEPVLTYINQQGLYR-- 211
                *::  *:     :.     :  :   .  ** : .**
```

Figure S6. Complementation of *E. coli* NadD with PfNMNAT.
E. coli containing *nadD::cam* and pBAD PfNMNAT were grown in the presence of arabinose to lag phase and then diluted into just LB or LB containing 0.2% arabinose (inducer) or 0.05% fucose (negative regulator). Growth was monitored continuously by absorbance at 600 nm for 10 hours. Error is provided as the SD of three independent biological replicates.

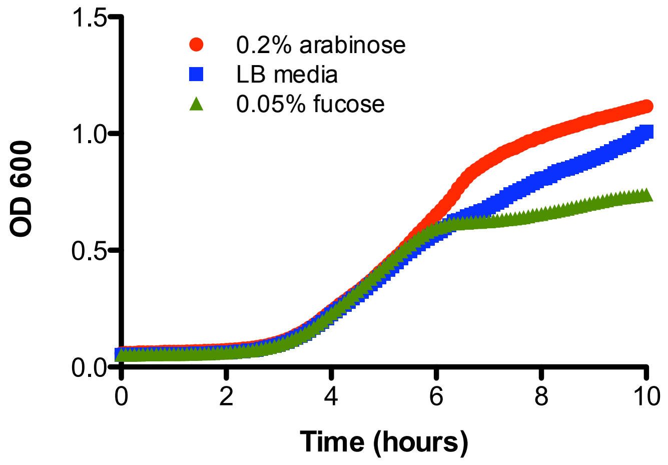

Figure S7. Alignment of PfNMNAT and the Human NMNAT homologs.
ClustalW2 was used to generate the alignment between the *P. falciparum* NMNAT (NCBI Gene ID: 814129) and the three *Homo sapiens* NMNATs: NMNAT1 (NCBI Gene ID: 64802), NMNAT2 (NCBI Gene ID:23057), NMNAT3 (NCBI Gene ID:349565).

```
HsNMNAT1        MENSEKTEVVLLACGSFNPITNMHLRLFELAKDYMNGTGRYTVVKGIISPVGDAYKKKGL 60
HsNMNAT3        ---------------------------------------MYQVIQGIISPVNDTYGKKDL 21
HsNMNAT2        MTETTKTHVILLACGSFNPITKGHIQMFERARDYLHKTGRFIVIGGIVSPVHDSYGKQGL 60
PfNMNAT         -----MHKNICIYGGSFDPITYAHEMVLDKISN-------LNWIHEIWVVICRCRNDKSL 48
                                                           :  *   :     .:.*

HsNMNAT1        IPAYHRVIMAELATKNSKWVEVDTWESLQKEWKETLKVLRHHQEKLEASD--CDHQQNSP 118
HsNMNAT3        AASHHRVAMARLALQTSDWIRVDPWESEQAQWMETVKVLRHHHSKLLRS---------PP 72
HsNMNAT2        VSSRHRLIMCQLAVQNSDWIRVDPWECYQDTWQTTCSVLEHHRDLMKRVTGCILSNVNTP 120
PfNMNAT         TEFHHRHNMFTIIINNSSKIIKS---------KIFLKDLESHSEMTPTYD---------- 89
                    **  *  :  :.*. :  .             . *. * .

HsNMNAT1        TLERPGRKRKWTETQDSSQKKSLEPKTKAVP----------------------------- 149
HsNMNAT3        QMEGP------------DHGKALFSTPAAVP----------------------------- 91
HsNMNAT2        SMTPVIGQPQNETPQPIYQNSNVATKPTAAKILGKVGESLSRICCVRPPVERFTFVDENA 180
PfNMNAT         ---------------LLKTQKELHPN---------------------------------- 100
                                    . : ..

HsNMNAT1        -------------KVKLLCGADLLESFAVPNLWKSEDITQIVANYGLICVTRAGNDAQKF 196
HsNMNAT3        -------------ELKLLCGADVLKTFQTPNLWKDAHIQEIVEKFGLVCVGRVGHDPKGY 138
HsNMNAT2        NLGTVMRYEEIELRILLLCGSDLLESFCIPGLWNEADMEVIVGDFGIVVVPRDAADTDRI 240
PfNMNAT         ------------YTFYFGLGSDLICDIFS---WDEG--EKLVLENAFIIIERG-----HF 138
                              . :  *:*::  :     *..     :* . .:: : *

HsNMNAT1        IYESDVLWKHRSNIHVVNEWIAN---DISSTKIRRALRRGQ--SIRYLVPDLVQEYIEKH 251
HsNMNAT3        IAESPILRMHQHNIHLAKEPVQN---EISATYIRRALGQGQ--SVKYLIPDAVITYIKDH 193
HsNMNAT2        MNHSSILRKYKNNIMVVKDDINHPMSVVSSTKSRLALQHGDG-HVVDYLSQPVIDYILKS 299
PfNMNAT         KIDESILKKFPKYYLINIPKLSFINFISSSEARKFLTKENDINDIKKYIHPLTIDYIIKY 198
                  .. :*  .     :    :       *:   :    ..:   :   :   .  ** .

HsNMNAT1        NLYSSESEDRNAGVILAPLQRNTAEAKT- 279
HsNMNAT3        GLYTKGSTWKGK-------STQSTEGKTS 215
HsNMNAT2        QLYINASG--------------------- 307
PfNMNAT         NLYDFN----------------------- 204
                 **
```

Supplemental Materials and Methods

Table S1. Primers Used in This Study

**PFC0910w primers**	
**pDC2 CAM GFP F**	GCG CGC CCT AGG ATG AAA TGC CTT GTT ATA GTT GAT G
**pDC2 CAM GFP R**	GCG CGC AGA TCT TGA CAA AAG TTT TGA TGA GTT AAT AAA
**PF13_0159 primers**	
**pPROEX F**	GCG CGC CCA TGG ATG CAT AAG AAT ATA TGT ATA TAT G
**pPROEX R**	GCG CGC CGT ACG ATT AAA ATC ATA TAA GTT ATA CTT TAT
**pDC2 CAM GFP F**	GCG CGC GGA TCC ATT AAA ATC ATA TAA GTT ATA CTT TAT
**pDC2 CAM GFP R**	GCG CGC CCT AGG ATG CAT AAG AAT ATA TGT ATA TAT G
**D110A SENSE**	TAC TTT GGT CTT GGA TCA GCT TTG ATA TGT GAT
**D110A ANTISENSE**	AAA TAT ATC ACA TAT CAA AGC TGA TCC AAG ACC
**PFF1410c primers**	
**pDC2 CAM GFP F**	GCG CGC CCT AGG ATG CAA GGT AAC AGG GAA AAC
**pDC2 CAM GFP R**	GCG CGC GGA TCC TTG ATT TAT GTG AGA ATT TTT TAT G
**PFI1310w primers**	
**pDC2 CAM GFP F**	GCG CGC CCT AGG ATG ATG AAT AAT ATC GGA TTA AGT TG
**pDC2 CAM GFP R**	GCG CGC GGA TCC TAA GTT CAA TTT TTT CTT CAA AGC G
**NadD primers**	
**ecNadD pBAD F**	GCG CGC TCT AGA AGG AGG AAT TAA CCA TGA AAT CTT TAC AGG CTC TGT
**ecNadD pBAD R**	GCG CGC AAG CTT TCA GCG ATA CAA GCC TTG TTG
**CamKanNadD F**	GTG GAA ACG CTG GCG AAT TTG ATT GGT CTG ACG CGG GTC ACA ATC ATC CCT TGT AGG CTG GAG CTG CTT CG
**CamKanNadD R**	CCA GTC GCC AAA AAA CAT TTC GTT GAG TTC AGG TAT GAT TTG CAC GGG GAG CAT ATG AAT ATC CTC CTT AG

Table S2. Strains Used in This Study

***E. coli* K-12 strains**	
**MC4100**	F- *araD139 (argF-lac)U169 rpsL150 relA1 flb5301 deoC1 ptsF25 thi*
**JO1**	MC4100 *ybeT::kan*
**JO2**	JO1 *nadD::cam*
**JO3**	MC4100 pBad-NadD
**JO4**	MC4100 pProEX-PF13_0159
**JO5**	MC4100 pProEX-PF13_0159^{D110A}
**JO6**	MC4100 pProEX-empty

Synthesis of 1a-a and derivatives

1a-a, (E)-4-(2-(anthracen-9-ylmethylene)hydrazinyl)-N-(4-bromophenyl)-4-oxobutanamide

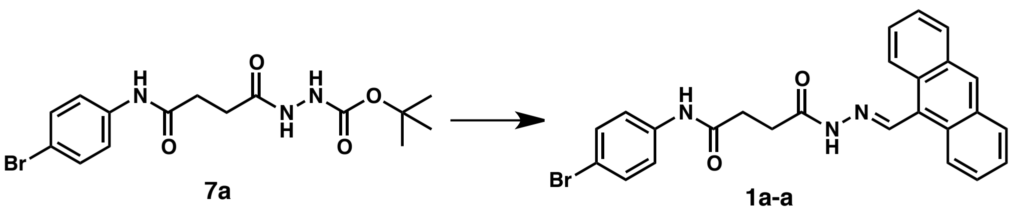

Intermediate **7a** (97.0 mg, 0.2523 mmol) was suspended in 10 ml of DCM and stirred. To this was added 5 ml of TFA. After 15 minutes the starting material dissolved as it was consumed, generating **3**.9-anthraldehyde (49.5 mg, 0.2400 mmol) was then added in one portion, producing a dark red solution. After five minutes, the solvent was removed under vacuum and the resulting oil was triturated with methanol and filtered, affording the yellow solid **1a-a** (95.1 mg, 0.20 mmol, 79.79 % over two steps), yellow solid, mp 268-270 °C; ^{1}H NMR (500 MHz, DMSO) δ 11.76/11.50 (s, 1H), 10.19/10.15 (s, 1H), 9.35/9.22 (s, 1H), 8.72 (s, 1H), 8.62 (t, *J* = 7.4 Hz, 2H), 8.16 (d, *J* = 8.4 Hz, 2H), 7.60 (m, 6H), 7.47 (t, *J* = 9.4 Hz, 2H), 3.01 (t, *J* = 6.7 Hz, 2H), 2.70 (m, 2H); ^{13}C NMR (125 MHz, DMSO) δ 173.52, 170.74, 170.56, 167.98, 144.65, 141.67, 138.81, 138.73, 131.54, 130.97, 129.53, 129.10, 127.25, 125.63, 125.23, 124.81, 124.63, 120.77, 31.11, 30.56, 29.08, 27.30; HRMS (m/z): $[M]^+$ calc. for $C_{25}H_{20}BrN_3O_2$, 473.07389; found 473.07351.

1a-c, (E)-4-(2-([1,1'-biphenyl]-2-ylmethylene)hydrazinyl)-N-(4-bromophenyl)-4-oxobutanamide

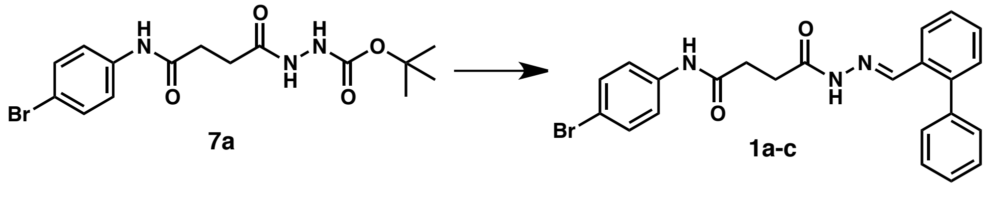

Synthesis followed that of **1a-a** using 46.8 mg (0.25 mmol) of [1,1'-biphenyl]-2-carbaldehyde. White solid (16 mg, 0.036 mmol, 14% over two steps). mp 206-208 °C; ^{1}H NMR (500 MHz, DMSO) δ 11.43/11.18 (s, 1H), 10.15 (s, 1H), 8.06/**7**.97 (s, 1H), 8.00 (t, *J* = 5.1 Hz, 1H), 7.56 (t, *J* = 9.9 Hz, 1H), 7.47 (m, 15H), 7.34 (m, 2H), 2.94 (t, *J* = 6.8 Hz, 1H), 2.62 (t, *J* = 6.8 Hz, 2H), 2.44 (t, *J* = 6.8 Hz, 1H); ^{13}C NMR (125 MHz, DMSO) δ 173.33, 170.76, 170.48, 167.76, 144.58, 143.86, 141.91, 141.61, 141.35, 139.09, 138.80, 131.56, 131.48, 130.42, 129.71, 129.66, 129.55, 128.55, 128.45, 127.77, 127.57, 125.45, 120.77, 114.63, 31.06, 30.58, 28.89, 27.33; HRMS (m/z): $[M]^+$ calc. for $C_{23}H_{20}BrN_3O_2$, 449.07389; found 449.07561.

1a-d, (*E*)-*N*-(4-bromophenyl)-4-(2-((2,3-dimethoxynaphthalen-1-yl)methylene)hydrazinyl)-4-oxobutanamide

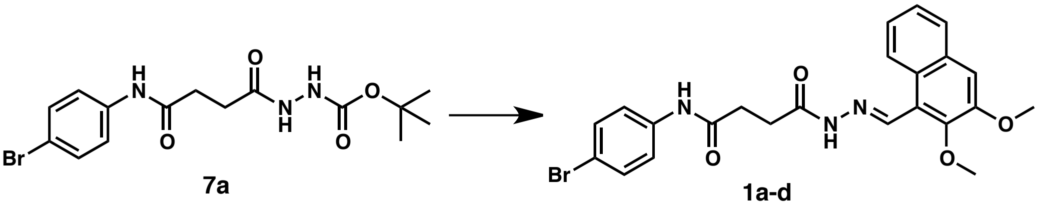

Synthesis followed that of **1a-a** usingusing54.0 mg (0.25 mmol) 2,3-dimethoxy-1-naphthaldehyde . Light pink solid (120.0 mg, 0.24 mmol, 99% over two steps), mp 315-317 °C; ^{1}H NMR (500 MHz, DMSO) δ 11.63/11.40 (s, 1H), 10.19/10.17 (s, 1H), 9.21/9.00 (d, *J* = 8.4 Hz, 1H), 8.77/8.66 (s, 1H), 7.84 (t, *J* = 7.5 Hz, 1H), 7.58 (dd, *J* = 8.9, 2.1 Hz, 3H), 7.53 (s, 1H), 7.45 (m, 4H), 3.96 (s, 3H), 3.89/3.79 (s, 3H), 3.00 (t, *J* = 6.7 Hz, 1H), 2.69 (t, *J* = 6.2 Hz, 2H), 2.58 (t, *J* = 7.0 Hz, 1H). ^{13}C NMR (125 MHz, DMSO) δ 173.40, 170.76, 170.55, 167.87, 151.12, 151.03, 150.30, 150.16, 143.16, 140.57, 138.82, 138.73, 131.56, 131.29, 131.22, 127.35, 127.21, 125.44, 121.46, 121.36, 120.78, 114.46, 114.35, 110.32, 110.06, 61.61, 55.79, 31.13, 30.56, 28.95, 27.43, 2.61. HRMS (m/z): $[M]^+$ calc. for $C_{23}H_{22}BrN_3O_4$, 483.07937; found 483.07968.

1a-e, (*E*)-*N*-(4-bromophenyl)-4-(2-((1-methyl-1*H*-indol-3-yl)methylene)hydrazinyl)-4-oxobutanamide

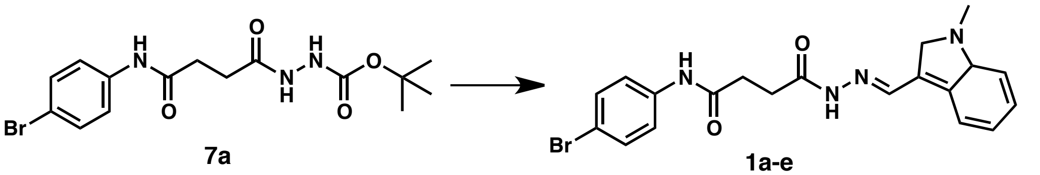

Synthesis followed that of **1a-a** using 40.3 mg (0.25 mmol) 1-methyl-1H-indole-3-carbaldehyde. After the addition of methanol, the flask was left in a freezer at 0° C overnight to allow crystal formation. Yellow solid (16.2 mg, 0.038 mmol, 15% over two steps), mp 224-225 °C; ^{1}H NMR (500 MHz, DMSO) δ 11.12/10.98 (s, 1H), 10.18/10.16 (s, 1H), 8.28/8.14 (s, 1H) 8.20/8.14 (d, *J* = 7.7 Hz, 1H), 7.77 (d, *J* = 5.4 Hz, 1H), 7.58 (m, 2H), 7.48 (m, 3H), 7.26 (t, *J* = 8.0 Hz, 1H), 7.17 (t, *J* = 8.0 Hz, 1H), 3.81 (s, 3H), 2.98 (t, *J* = 6.6 Hz, 2H), 2.68 (t, *J* = 6.6 Hz, 2H); ^{13}C NMR (125 MHz, DMSO) δ 172.61, 170.96, 169.02, 166.97, 142.30, 139.64, 138.88, 137.60, 133.75, 131.56, 124.50, 124.50, 122.71, 121.73, 120.78, 120.56, 114.33, 114.33, 110.67, 110.33, 30.58, 27.29; HRMS (m/z): $[M]^+$ calc. for $C_{20}H_{19}BrN_4O_2$, 426.06914; found 426.06852.

1b-a, (E)-4-(2-(anthracen-9-ylmethylene)hydrazinyl)-N-(3-bromophenyl)-4-oxobutanamide

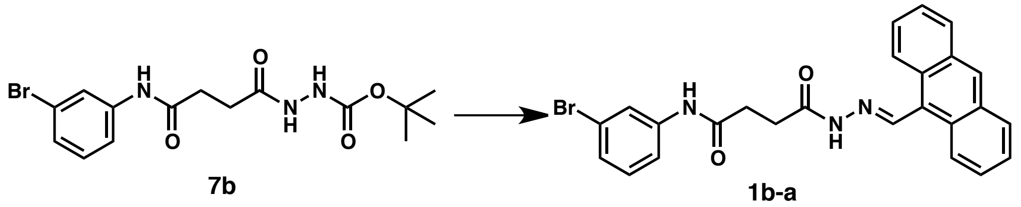

Synthesis followed that of **1a-a** usingusing205.3 mg (1.00 mmol) 9-anthraldehyde and **7b** in place of **7a**. Yellow solid (237.8 mg, 0.50 mmol, 50% over two steps), mp 234-236 °C; ^{1}H NMR (500 MHz, DMSO) δ 11.76/11.51 (s, 1H), 10.23/10.19 (s, 1H), 9.36/9.22 (s, 1H), 8.72 (s, 1H), 8.62 (t, *J* = 7.7 Hz, 2H), 8.16 (d, *J* = 8.4 Hz, 2H), 7.99 (d, *J* = 11.8 Hz, 1H), 7.64 (m, 2H), 7.58 (m, 2H), 7.49 (t, *J* = 9.3 Hz, 1H), 7.25 (t, *J* = 8.3 Hz, 1H), 7.20 (d, *J* = 8.0 Hz, 1H), 3.01 (t, *J* = 6.7 Hz, 1H), 2.70 (m, 3H); ^{13}C NMR (125 MHz, DMSO) δ 174.55, 173.48, 170.98, 170.16, 144.66, 141.69, 140.99, 130.97, 130.81, 129.51, 129.32, 129.09, 127.24, 125.62, 125.23, 124.81, 124.62, 121.62, 121.22, 117.58. HRMS (m/z): $[M]^+$ calc. for $C_{25}H_{20}BrN_3O_2$, 473.07389; found 473.07375.

1d-a , (E)-4-(2-(anthracen-9-ylmethylene)hydrazinyl)-N-(4-hydroxyphenyl)-4-oxobutanamide

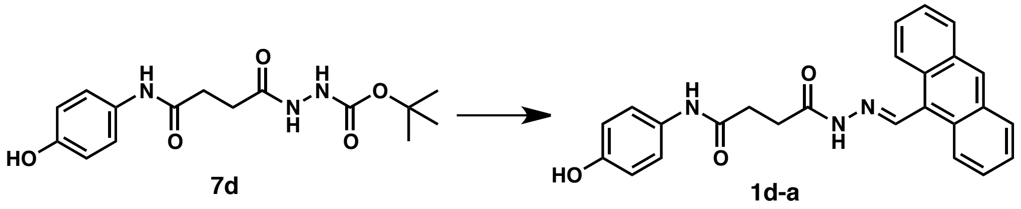

Synthesis followed that of **1a-a** using 108.0 mg (0.52 mmol) 9-anthraldehyde and **7d** in place of **7a**. Yellow solid (44.0 mg, 0.11 mmol, 21% over two steps), mp 249-251 °C; ^{1}H NMR (500 MHz, DMSO) δ 11.74/11.48 (s, 1H), 9.78/9.72 (s, 1H), 9.35/9.21 (s, 1H), 9.14/9.11 (s, 1H), 8.72 (s, 1H), 8.62 (dd, *J* = 8.5, 6.1 Hz, 2H), 8.16 (d, *J* = 8.5 Hz, 2H), 7.63 (t, *J* = 7.5 Hz, 2H), 7.58 (t, *J* = 7.5 Hz, 2H), 7.37 (dd, *J* = 11.8, 9.2 Hz, 2H), 6.67 (t, *J* = 9.2 Hz, 2H), 2.99 (t, *J* = 6.9 Hz, 1H), 2.65 (m, 3H); ^{13}C NMR (125 MHz, DMSO) δ 173.65, 169.67, 169.50, 168.12, 153.00, 144.61, 141.57, 131.20, 130.98, 129.52, 129.30, 129.09, 127.24, 125.63, 125.26, 124.65, 120.61, 115.02, 30.96, 30.35, 29.42, 27.49; HRMS (m/z): $[M]^+$ calc. for $C_{25}H_{21}N_3O_3$, 411.15829; found 411.15969.

1e-a, (E)-4-(2-(anthracen-9-ylmethylene)hydrazinyl)-N-(3-hydroxyphenyl)-4-oxobutanamide

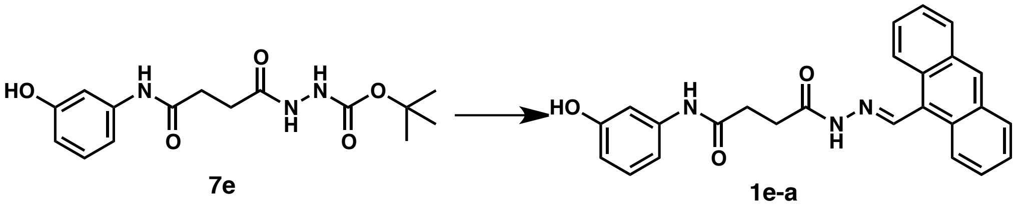

Synthesis followed that of **1a-a** using 25.5 mg (0.12 mmol) 9-anthraldehyde and **7e** in place of **7a**. Yellow solid (19.2 mg, 0.046 mmol, 37% over two steps), mp 248-250 °C; ^{1}H NMR (500 MHz, DMSO) δ 11.75/11.49 (s, 1H), 9.90/9.86 (s, 1H), 9.34 (m, 1H), 9.22 (s, 1H), 8.72 (s, 1H), 8.62 (dd, *J* = 8.5, 3.9 Hz, 2H), 8.16 (d, *J* = 8.5 Hz, 2H), 7.63 (m, 2H), 7.58 (t, *J* = 8.5 Hz, 2H), 7.18 (s, 1H), 7.04 (t, *J* = 8.2 Hz, 1H), 6.96 (dd, *J* = 12.5, 8.2 Hz, 1H), 6.41 (dd, *J* = 9.3, 7.0 Hz, 1H), 3.00 (t, *J* = 6.8 Hz, 1H), 2.66 (m, 3H); ^{13}C NMR (125 MHz, DMSO) δ 173.60, 170.35, 168.06, 157.59, 144.60, 141.58, 140.48, 131.03, 130.97, 130.68, 129.52, 129.51, 129.33, 129.09, 127.24, 125.62, 125.23, 124.63, 109.97, 109.66, 106.05, 66, 31.13, 30.55, 29.25, 27.36.. HRMS (m/z): $[M]^+$ calc. for $C_{25}H_{21}N_3O_3$, 411.15829; found 411.15863.

1f-a, (E)-4-(2-(anthracen-9-ylmethylene)hydrazinyl)-N-(2-hydroxyphenyl)-4-oxobutanamide

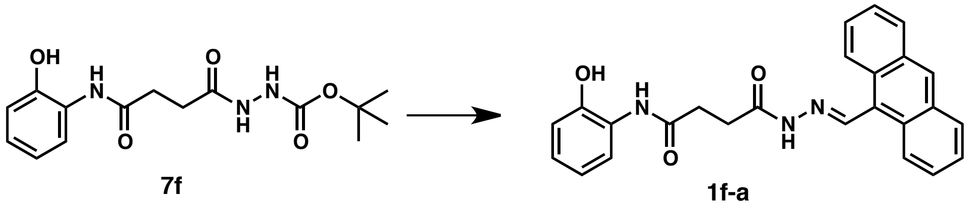

Synthesis followed that of **1a-a** using 25.8 mg (0.125 mmol) 9-anthraldehyde and **7f** in place of **7a**. Yellow solid (31.3 mg, 0.076 mmol, 61% over two steps), mp 260-262 °C; ^{1}H NMR (500 MHz, DMSO) δ 11.74/11.50 (s, 1H), 9.75/9.73 (s, 1H), 9.35/9.21 (s, 1H), 9.35/9.21 (s, 1H), 8.71 (s, 1H), 8.62 (t, *J* = 8.3 Hz, 2H), 8.16 (d, *J* = 8.3 Hz, 2H), 7.73 (dd, *J* = 16.3, 8.0 Hz, 1H), 7.63 (t, *J* = 8.3 Hz, 2H), 7.58 (t, *J* = 8.3 Hz, 3H), 6.93 (t, *J* = 7.9 Hz, 1H), 6.85 (t, *J* = 7.9 Hz, 1H), 6.75 (d, *J* = 7.9 Hz, 1H), 3.00 (t, *J* = 6.8 Hz, 1H), 2.79 (m, 2H), 2.64 (m, 1H). ^{13}C NMR (125 MHz, DMSO) δ 173.59, 171.10, 146.48, 144.58, 141.59, 131.00, 129.53, 129.30, 129.08, 127.24, 126.51, 125.62, 125.23, 124.82, 124.64, 122.11, 118.98, 115.79, 109.58, 31.21, 30.30, 29.45, 27.56. HRMS (m/z): $[M]^+$ calc. for $C_{25}H_{21}N_3O_3$, 411.15829; found 411.15927.

1g-a, (E)-4-(2-(anthracen-9-ylmethylene)hydrazinyl)-N-(4-hydroxyphenyl)-4-oxobutanamide

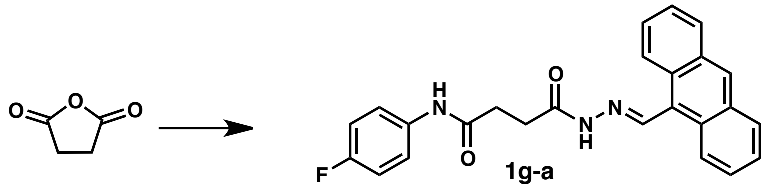

Tert-butyl carbazate (660.6 mg, 5.0 mmol) was added to a solution of succinic anhydride (501.8 mg, 5.0 mmol) in 2.5 mL THF in one portion, to form acid **6**. To this solution was added 4-fluoroaniline (555.7 mg, 5.0 mmol) in one portion followed by 1.1 ml of N,N'-diisopropylcarbodiimide (DIC) after five minutes. After fifteen minutes the reaction was diluted with 7.5 ml of THF and TFA (12.5 ml) was added and allowed to react for 10 minutes. 9-anthraldehyde (555.7 mg, 5.0 mmol) was added and allowed to react for 20 minutes. The solvent was then removed under vacuum and the resulting material triturated with methanol, yielding a bright orange solid (1.0082 g, 2.439 mmol, 48.77 % over four steps), mp 225-226 °C; ^{1}H NMR (500 MHz, DMSO) δ 11.75/11.49 (s, 1H), 10.11/10.06 (s, 1H), 9.35/9.22 (s, 1H), 8.72 (s, 1H), 8.62 (m, 2H), 8.16 (d, *J* = 8.3 Hz, 2H), 7.61 (m, 6H), 7.13 (q, *J* = 9.2 Hz, 2H), 3.01 (t, *J* = 6.8 Hz, 1H), 2.70 (m, 3H); ^{13}C NMR (125 MHz, DMSO) δ 173.43, 170.42, 170.21, 167.90, 158.68, 156.78, 151.12, 150.16, 143.15, 140.54, 135.79, 131.30, 131.30, 131.23, 127.36, 125.68, 125.45, 125.25, 121.47, 120.54, 115.37, 115.19, 110.06, 61.62, 55.80, 31.03, 30.42, 29.05, 27.49. HRMS (m/z): $[M]^+$ calc. for C25H20FN3O2, 413.15396; found 413.15384.

1g-c, (E)-4-(2-([1,1'-biphenyl]-2-ylmethylene)hydrazinyl)-N-(4-fluorophenyl)-4-oxobutanamide

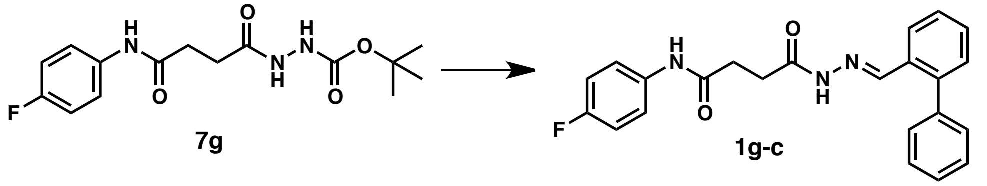

Synthesis followed that of **1a-a** using using44.0 mg (0.24 mmol) [1,1'-biphenyl]-2-carbaldehyde in place of 9-anthraldehyde and **7g** in place of **7a.** White solid (57.4 mg, 0.15 mmol, 62% over two steps). White solid, mp 193-194 °C; ^{1}H NMR (500 MHz, DMSO) δ 11.43/11.18 (s, 1H), 10.06 (s, 1H), 8.06/7.97 (s, 1H), 8.00 (m, 1H), 7.60 (ddd, *J* = 11.0, 9.1, 5.1 Hz, 2H), 7.48 (m, 5H), 7.35 (m, 2H), 7.12 (td, *J* = 9.1, 2.2 Hz, 2H), 2.94 (t, *J* = 6.9 Hz, 1H), 2.61 (dt, *J* = 7.0, 3.5 Hz, 2H), 2.45 (t, *J* = 6.9 Hz, 1H). ^{13}C NMR (125 MHz, DMSO) δ 173.36, 170.42, 170.15, 167.80, 143.87, 141.91, 141.61, 141.33, 139.09, 135.86, 135.75, 131.57, 131.49, 130.43, 129.65,

128.59, 127.78, 127.58, 125.46, 120.58, 115.37, 115.17, 30.96, 30.46, 28.99, 27.40. HRMS (m/z): $[M]^+$ calc. for $C_{23}H_{20}FN_3O_2$, 389.15396; found 389.15506.

1g-d, (*E*)-*N*-(4-fluorophenyl)-4-(2-((2,3-dimethoxynaphthalen-1-yl)methylene)hydrazinyl)-4-oxobutanamide.

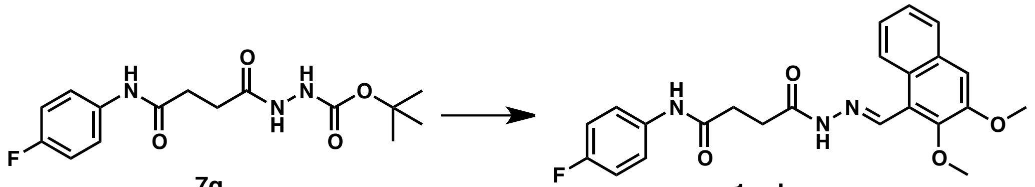

7g 1g-d

Synthesis followed that of **1a-a** using 53.4 mg (0.25 mmol) 2,3-dimethoxy-1-naphthaldehyde in place of 9-anthraldehyde and **7g** in place of **7a**. Salmon-colored solid (89.9 mg, 0.21 mmol, 85% over two steps). mp 189-192 °C; ^{1}H NMR (500 MHz, DMSO) δ 11.63/11.39 (s, 1H), 10.11/10.08 (s, 1H), 9.21/9.01 (d, *J* = 7.8 Hz, 1H), 8.77/8.66 (s, 1H), 7.84 (td, *J* = 7.4, 1.5 Hz, 1H), 7.62 (ddt, *J* = 8.0, 5.1, 2.5 Hz, 2H), 7.53 (s, 1H), 7.43 (m, 2H), 7.13 (td, *J* = 8.0, 3.3 Hz, 2H), 3.96 (s, 3H), 3.85 (d, *J* = 9.3 Hz, 3H), 3.00 (t, *J* = 6.7 Hz, 1H), 2.68 (t, *J* = 6.5 Hz, 2H), 2.59 (t, *J* = 6.9 Hz, 1H); ^{13}C NMR (125 MHz, DMSO) δ 173.43, 170.42, 170.22, 167.90, 158.68, 156.78, 151.12, 150.17, 143.15, 140.54, 135.80, 131.30, 127.36, 125.71, 125.52, 125.45, 125.30, 121.47, 120.54, 115.37, 115.21, 110.06, 61.62, 55.80, 31.02, 30.42, 29.05, 27.49; HRMS (m/z): $[M]^+$ calc. for $C_{23}H_{22}FN_3O_4$, 423.15943; found 423.15954.

1g-e, (*E*)-*N*-(4-bromophenyl)-4-(2-((1-methyl-1*H*-indol-3-yl)methylene)hydrazinyl)-4-oxobutanamide

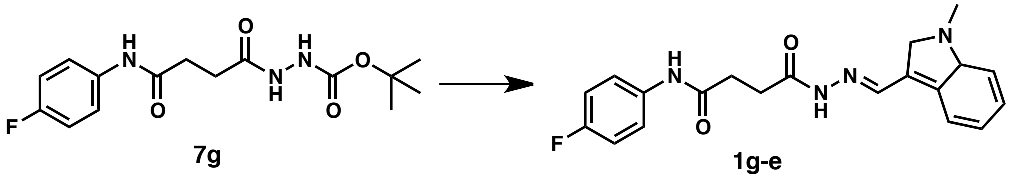

7g 1g-e

Synthesis followed that of **1a-a** using 38.2 mg (0.24 mmol) 1-methyl-1H-indole-3-carbaldehyde in place of 9-anthraldehyde and **7g** in place of **7a**. Yellow solid (60.4 mg, 0.16 mmol, 67% over two steps). Pale yellow solid, mp 220-223 °C; ^{1}H NMR (500 MHz, DMSO) δ 11.12/10.97 (s, 1H), 10.09/10.07 (s, 1H), 8.28/8.14 (s, 1H), 8.20/8.14 (d, *J* = 7.5 Hz, 1H), 7.77 (d, *J* = 5.6 Hz, 1H), 7.62 (dd, *J* = 8.3, 4.8 Hz, 2H), 7.50 (d, *J* = 8.2 Hz, 1H), 7.26 (m, 1H), 7.17 (t, *J* = 7.5 Hz, 1H), 7.13 (t, *J* = 8.3, 2H), 3.81 (s, 3H), 2.98 (t, *J* = 6.7 Hz, 1H), 2.66 (dt, *J* = 14.2, 6.7 Hz, 2H); ^{13}C NMR (125 MHz, DMSO) δ 172.63, 170.60, 170.30, 166.99, 156.77, 142.28, 139.60, 137.59, 137.51, 135.95, 133.75, 124.69, 124.50, 122.69, 122.09, 121.71, 120.74, 120.72, 120.54, 115.38,

115.18, 110.61, 110.32, 31.31, 30.45, 29.18, 27.35, 12.10, -2.62; HRMS (m/z): $[M]^+$ calc. for C20H19FN4O2, 366.14920; found 366.15032.

2a-a, (*E*)-5-(2-(anthracen-9-ylmethylene)hydrazinyl)-*N*-(4-bromophenyl)-5-oxopentanamide.

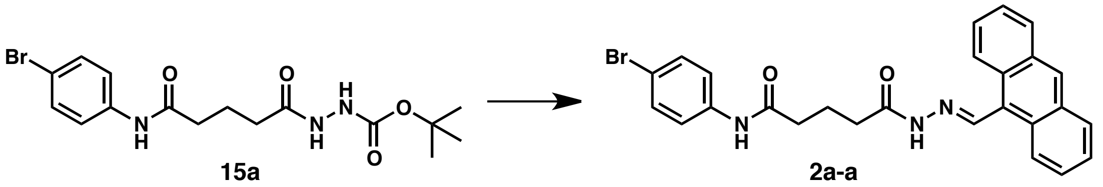

Synthesis followed that of **1a-a** using 101.1 mg (0.49 mmol) 9-anthraldehyde and with **15a** in place of **7a**. Yellow solid (175.2 mg, 0.36 mmol, 72% over two steps), mp 245-249 °C; ^{1}H NMR (500 MHz, DMSO) δ 11.66/11.48 (d, *J* = 89.6 Hz, 1H), 10.02/9.95 (d, *J* = 35.9 Hz, 1H), 9.35/9.20 (s 1H), 8.71 (d, *J* = 12.0 Hz, 1H), 8.63 (d, *J* = 9.0 Hz, 1H), 8.56 (d, *J* = 8.2 Hz, 1H), 8.15 (t, *J* = 7.2 Hz, 2H), 7.57 (m, 5H), 7.45 (dd, *J* = 27.9, 8.1 Hz, 2H), 2.74 (t, *J* = 7.3 Hz, 1H), 2.42 (m, 3H), 1.96 (m, 2H); ^{13}C NMR (125 MHz, DMSO) δ 173.92, 171.10, 171.04, 168.29, 144.89, 141.56, 138.66, 131.47, 131.04, 130.94, 129.53, 129.48, 129.29, 129.06, 128.40, 127.18, 126.90, 125.57, 125.16, 124.83, 124.57, 120.93, 114.47, 35.77, 35.60, 33.50, 31.38, 20.77, 20.15; HRMS calc. for $C_{26}H_{22}BrN_3O_2$ $[M]^+$: 487.08954, found: 487.08908.

2a-c, (*E*)-5-(2-([1,1'-biphenyl]-2-ylmethylene)hydrazinyl)-*N*-(4-bromophenyl)-5-oxopentanamide.

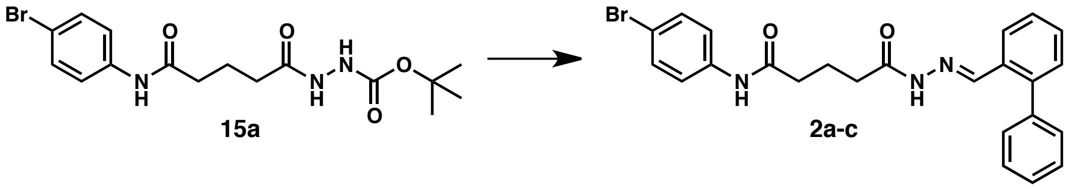

Synthesis followed that of **1a-a** using 87.4 mg (0.48 mmol) [1,1'-biphenyl]-2-carbaldehyde in place of 9-anthraldehyde and **15a** in place of **7a**. After the addition of methanol, the flask was left in a freezer at 0° C overnight to allow crystal formation. Yellow solid (166.5 mg, 0.36 mmol, 75% over two steps), mp 125-126 °C; ^{1}H NMR (500 MHz, DMSO) δ 11.32/11.13 (s, 1H), 10.04 (s, 1H), 8.06/7.93 (s, 1H), 8.01/7.92 (d, *J* = 7.7 Hz, 2H), 7.56 (t, *J* = 16.5 Hz, 2H), 7.46 (m, 6H), 7.32 (m, 3H), 2.66 (t, *J* = 7.1 Hz, 1H), 2.39 (t, *J* = 7.1 Hz, 1H), 2.33 (t, *J* = 7.1 Hz, 1H), 2.18 (t, *J* = 7.1 Hz, 1H), 1.86 (dp, *J* = 21.1, 7.1 Hz, 2H); ^{13}C NMR (125 MHz, DMSO) δ 173.88, 171.20, 170.96, 168.04, 143.99, 141.94, 141.57, 141.25, 139.09, 139.02, 138.72, 131.54, 131.51, 131.47,

130.40, 129.63, 128.53, 127.78, 127.67, 127.55, 125.43, 120.92, 114.50, 35.72, 35.51, 33.27, 31.24, 20.71, 20.19. HRMS (m/z): $[M]^+$ calc. for $C_{24}H_{22}BrN_3O_2$, 463.08954; found 463.08998.

2a-d, (*E*)-*N*-(4-bromophenyl)-5-(2-((2,3-dimethoxynaphthalen-1-yl)methylene)hydrazinyl)-5-oxopentanamide.

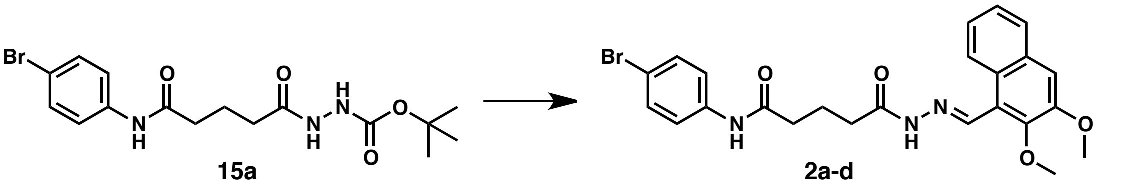

Synthesis followed that of **1a-a** using 103.54 mg (0.47 mmol) 2,3-dimethoxy-1-naphthaldehyde in place of 9-anthraldehyde and **15a** in place of **7a**. Pale yellow solid (171.0 mg, 0.34 mmol, 71% over two steps), mp 211-213 °C; 1H NMR (500 MHz, DMSO) δ 11.53/11/35 (s, 1H), 10.08/10.05 (s, 1H), 9.23/8.91 (d, *J* = 8.5 Hz, 1H), 8.77/8.64 (d, *J* = 65.2 Hz, 1H), 7.83 (t, *J* = 6.8 Hz, 1H), 7.56 (m, 6.5H), 7.50/7.32 (t, *J* = 7.8 Hz, 1H), 3.95 (d, *J* = 3.2 Hz, 3H), 3.83 (d, *J* = 14.9 Hz, 3H), 2.73 (t, *J* = 7.2 Hz, 1H), 2.42 (dt, *J* = 13.0, 7.2 Hz, 2H), 2.32 (t, *J* = 7.2 Hz, 1H), (dp, *J* = 7.2, 7.9 Hz, 2H); ^{13}C NMR (125 MHz, DMSO) δ 173.83, 171.09, 168.18, 151.08, 151.02, 150.32, 150.11, 143.33, 140.43, 138.71, 131.53, 131.46, 131.23, 127.30, 125.71, 125.56, 125.45, 125.40, 125.24, 121.41, 120.94, 114.52, 114.48, 110.02, 109.56, 61.57, 55.79, 35.79, 35.57, 33.33, 31.50, 20.80, 20.13. HRMS (m/z): $[M]^+$ calc. for $C_{24}H_{24}BrN_3O_4$, 497.09502; found 497.09641.

2a-e, (*E*)-*N*-(4-bromophenyl)-5-(2-((1-methyl-1*H*-indol-3-yl)methylene)hydrazinyl)-5-oxopentanamide

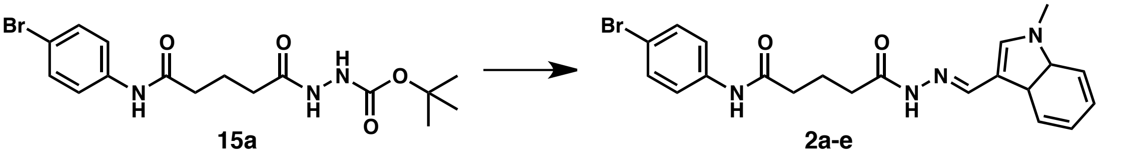

Synthesis followed that of **1a-a** using 78.9 mg (0.49 mmol) 1-methyl-1H-indole-3-carbaldehyde in place of 9-anthraldehyde and 1**5a** in place of **7a**. After the addition of methanol, the flask was left in a freezer at 0° C overnight to allow crystal formation.. Pale yellow solid (143.6 mg, 0.33 mmol, 66% over two steps), mp 218-219 °C; 1H NMR (500 MHz, DMSO) δ 11.02/10.92 (s, 1H), 10.07 (s, 1H), 8.28/8.12 (s, 1H), 8.21/8.07 (d, *J* = 7.7 Hz, 2H), 7.74 (d, *J* = 17.7 Hz, 1H), 7.58 (d, *J* = 8.3 Hz, 2H), 7.45 (dd, *J* = 13.9, 8.3 Hz, 3H), 7.24 (dd, *J* = 17.8, 8.0 Hz, 1H), 7.12 (dt, *J* = 48.5, 8.0 Hz, 2H), 3.80 (d, *J* = 7.2 Hz, 3H), 2.72 (t, *J* = 7.1 Hz, 1H), 2.44 (t, *J* = 7.1 Hz, 1H), 2.38 (t, *J* = 7.1 Hz, 1H), 2.24 (t, *J* = 7.1 Hz, 1H), 1.92 (dt, *J* = 20.4, 7.1 Hz, 2H); ^{13}C NMR

(125 MHz, DMSO) δ 173.08, 171.21, 171.06, 167.32, 142.40, 138.72, 137.55, 133.65, 131.48, 124.44, 122.61, 122.03, 121.54, 120.92, 120.69, 114.45, 110.57, 110.21, 35.93, 35.65, 33.36, 31.35, 20.99, 20.16; HRMS (m/z): $[M]^+$ calc. for $C_{21}H_{21}BrN_4O_2$, 440.08479; found 440.08379.

2g-a, (*E*)-5-(2-(anthracen-9-ylmethylene)hydrazinyl)-*N*-(4-fluorophenyl)-5-oxopentanamide.

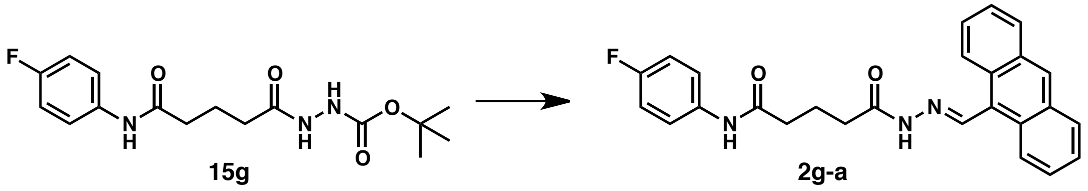

Synthesis followed that of **1a-a** using 101.0 mg (0.49 mmol) 9-anthraldehyde and 15**g** (281.5 mg, 0.51 mmol) in place of **7a**. After the addition of methanol, the flask was left in a freezer at 0° C overnight to allow crystal formation. Yellow solid (162.8 mg, 0.38 mmol, 78% over two steps), mp 210-212 °C; ^{1}H NMR (500 MHz, DMSO) δ 11.75/11.49 (s, 1H), 10.03/9.97 (s, 1H), 9.36/9.21 (s, 1H), 8.72 (d, *J* = 11.1 Hz, 1H), 8.64 (d, *J* = 8.8 Hz, 1H), 8.57 (d, *J* = 8.1 Hz, 1H), 8.16 (t, *J* = 7.6 Hz, 2H), 7.64 (m, 3H), 7.58 (q, *J* = 7.6 Hz, 3H), 7.12 (dtd, *J* = 26.3, 9.1, 2.7 Hz, 2H), 2.75 (t, *J* = 7.3 Hz, 2H), 2.42 (dq, *J* = 15.7, 7.3 Hz, 2H), 1.97 (h, *J* = 7.3 Hz, 2H); ^{13}C NMR (125 MHz, DMSO) δ 173.93, 170.77, 170.71, 168.30, 144.87, 141.56, 135.72, 130.94, 129.53, 129.49, 129.28, 129.05, 128.40, 127.17, 126.89, 125.49, 125.19, 124.78, 124.68, 124.54, 120.75, 115.31, 115.22, 115.17, 115.14, 35.68, 35.50, 33.53, 31.42, 20.85, 20.22; HRMS (m/z) $[M]^+$ calc. for $C_{25}H_{20}BrN_3O_2$, 427.16961, found: 427.16890.

2g-c, (*E*)-5-(2-([1,1'-biphenyl]-2-ylmethylene)hydrazinyl)-*N*-(4-fluorophenyl)-5-oxopentanamide

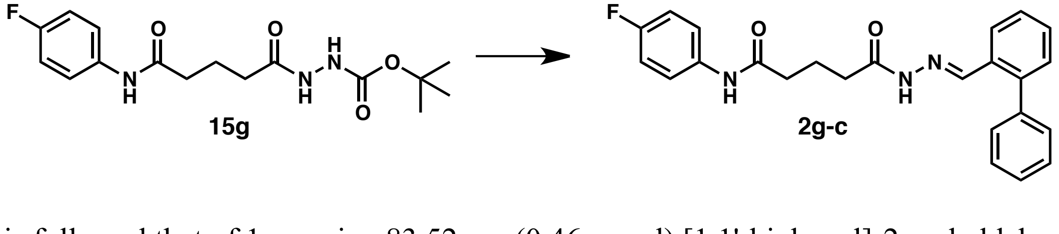

Synthesis followed that of 1a-a using 83.52 mg (0.46 mmol) [1,1'-biphenyl]-2-carbaldehyde in place of 9-anthraldehyde and 15g (282.7 mg, 0.514 mmol) in place of 7a. After the addition of methanol, the flask was left in a freezer at 0° C overnight to allow crystal formation. (44.9 mg, 0.11 mmol, 24% over two steps), mp 154-156 °C; ^{1}H NMR (500 MHz, DMSO) δ 11.32/11.13 (d, *J* = 95.0 Hz, 1H), 9.96 (s, 1H), 8.06/**7**.94 (s, 1H), 8.01/**7**.93 (d, *J* = 7.9 Hz, 1H), 7.60 (m, 2H),

7.47 (m, 4.4H (contains half of rotameric doublet)), 7.44/7.38 (t, 1H), 7.32 (m, 3H), 7.12 (t, *J* = 8.4 Hz, 2H), 2.66 (t, *J* = 7.0 Hz, 1H), 2.38 (t, *J* = 7.0 Hz, 1H), 2.32 (t, *J* = 7.0 Hz, 1H), 2.18 (t, *J* = 7.0 Hz, 1H), 1.87 (dt, *J* = 19.0, 7.0 Hz, 2H); ^{13}C NMR (125 MHz, DMSO) δ 173.88, 170.83, 170.64, 168.12, 158.75, 143.99, 141.92, 141.58, 141.25, 139.11, 139.09, 135.75, 135.72, 131.55, 131.47, 130.40, 129.73, 129.66, 129.55, 129.49, 128.54, 127.78, 127.72, 127.69, 127.65, 127.56, 125.44, 35.62, 35.42, 33.31, 31.28, 20.81, 20.29; HRMS (m/z): HRMS (m/z): $[M]^+$ calc. for $C_{24}H_{22}FN_3O_2$, 403.16961; found 403.16839.

2g-d, (*E*)-*N*-(4-fluorophenyl)-5-(2-((2,3-dimethoxynaphthalen-1-yl)methylene)hydrazinyl)-5-oxopentanamide

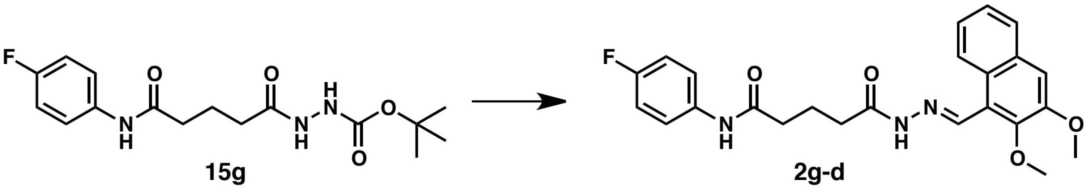

Synthesis followed that of **1a-a** using 103.9 mg (0.48 mmol) 2,3-dimethoxy-1-naphthaldehyde in place of 9-anthraldehyde and **15g** in place of **7a**. (110.6 mg, 0.25 mmol, 52% over two steps), mp 195-197°C; ^{1}H NMR (500 MHz, DMSO) δ 11.53/11.35 (s, 1H), 10.00/9.98 (s, 1H), 9.23/8.92 (d, *J* = 8.3 Hz, 1H), 8.77/8.64 (s, 1H), 7.83 (m, 2H), 7.60 (dd, *J* = 13.3, 7.9 Hz, 2H), 7.51 (d, *J* = 12.5 Hz, 1H), 7.43 (dt, *J* = 16.1, 8.2 Hz, 2H), 7.33 (t, *J* = 7.9 Hz, 2H), 7.11 (m, 3H), 3.95 (d, *J* = 3.6 Hz, 3H), 3.82 (d, *J* = 1.6 Hz, 3H), 2.73 (t, *J* = 7.1 Hz, 1H), 2.40 (dt, *J* = 14.1, 7.1 Hz, 3H), 2.32 (t, *J* = 7.1 Hz, 1H), 1.95 (p, *J* = 7.1 Hz, 2H); ^{13}C NMR (125 MHz, DMSO) δ 173.86, 170.79, 170.71, 168.21, 151.10, 150.12, 143.34, 140.38, 135.76, 131.23, 127.28, 126.32, 125.59, 125.13, 121.36, 120.71, 115.13, 109.99, 61.54, 55.74, 35.73, 35.48, 33.37, 31.59, 20.91, 20.21; HRMS (m/z): $[M]^+$ calc. for $C_{24}H_{24}FN_3O_4$, 437.17508; found 437.17546.

2g-e, (*E*)-*N*-(4-fluorophenyl)-5-(2-((1-methyl-1*H*-indol-3-yl)methylene)hydrazinyl)-5-oxopentanamide

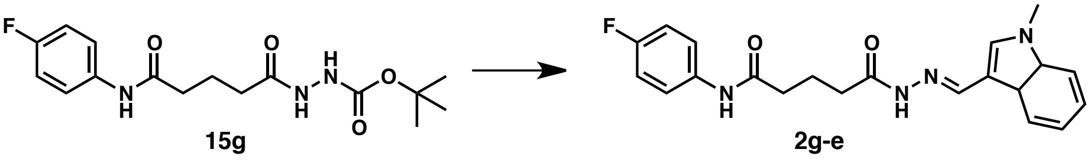

Synthesis followed that of **1a-a** using 79.3 mg (0.50 mmol) 1-methyl-1H-indole-3-carbaldehyde in place of 9-anthraldehyde and 1**5g** in place of **7a**. After the addition of methanol, the flask was left in a freezer at 0° C overnight to allow crystal formation. Pale yellow solid (140.5 mg, 0.37

mmol, 74% over two steps), mp 186-187 °C; 1H NMR (500 MHz, DMSO) δ 11.02/10.92 (s, 1H), 9.99 (s, 1H), 8.28/8.13 (s, 1H), 8.21/8.07 (d, *J* = 7.9 Hz, 2H), 7.74 (d, *J* = 16.0 Hz, 1H), 7.61 (m, 2H), 7.47 (d, *J* = 8.0 Hz, 1H), 7.16 (m, 4H), 3.80 (d, *J* = 7.6 Hz, 3H), 2.72 (t, *J* = 7.2 Hz, 1H), 2.43 (t, *J* = 7.2 Hz, 1H), 2.36 (t, *J* = 7.2 Hz, 1H), 2.24 (t, *J* = 7.2 Hz, 1H), 1.92 (dp, *J* = 21.3, 7.2 Hz, 2H); ^{13}C NMR (125 MHz, DMSO) δ 173.12, 170.91, 170.76, 167.38, 158.77, 142.47, 139.55, 137.57, 137.52, 135.80, 135.78, 133.69, 124.71, 124.47, 122.64, 122.10, 121.68, 120.71, 120.63, 120.58, 115.32, 115.24, 115.14, 110.68, 110.60, 110.26, 35.86, 35.54, 33.48, 31.42, 21.13, 20.25. HRMS (m/z): $[M]^+$ calc. for $C_{21}H_{21}FN_4O_2$, 380.16485; found 380.16467.

7a, tert-butyl 2-(4-((4-bromophenyl)amino)-4-oxobutanoyl)hydrazinecarboxylate

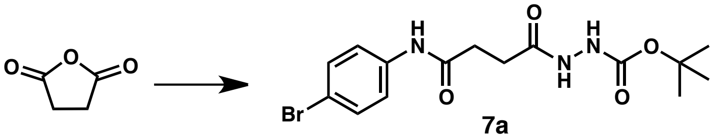

7a

Succinic anhydride (1.005 g, 10.03 mmol) and tert-butyl carbazate (1.3211 g, 10.00 mmol) were dissolved and stirred in THF (5 ml), resulting in the formation of intermediate **6** over ten minutes. 4-bromoaniline (1.875 g, 10.09 mmol) was added to the flask and the solution allowed to stirfor five minutes. DIC(1.7668 g, 2.2 ml, 14 mmol) was then added dropwise over five minutes affording white precipitate. The reaction mixture was diluted with 15 ml ethyl acetate and filtered, yielding white solid **7a** with 0.6 equivalents of N,N'-diisopropylurea. (919.63, 3.96 mmol, 40% over two steps). White olid, mp 190-192 °C; ^{1}H NMR (500 MHz, DMSO-d6) δ 10.12 (s, 1H), 9.59 (s, 1H), 8.74 (s, 1H), 7.56 (d, *J* = 8.8 Hz, 2H), 7.46 (d, *J* = 8.8 Hz, 2H), 2.55 (t, *J* = 7.1 Hz, 2H), 2.41 (t, *J* = 7.1 Hz, 2H), 1.38 (s, 9H). ^{13}C NMR (125 MHz, DMSO-d6) 171.05, 170.40, 155.32, 138.72, 131.55, 120.82, 114.46, 79.06, 31.21, 28.06, 23.38. MS (m/z): $[M]^+$ calc. for $C_{15}H_{20}BrN_3O_4$, 385.1; found 385.1.

7b, tert-butyl 2-(4-((3-bromophenyl)amino)-4-oxobutanoyl)hydrazinecarboxylate

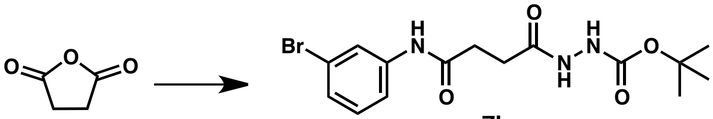

7b

Synthesis followed that of **7a** with 1.003 g (10.0 mmol) succinic anhydride and with 3-bromoaniline in place of 4-bromoaniline. After ethyl acetate trituration, the filtrate was rotovaped, and 15 ml diethyl ether added to the remaining solid. The flask was placed in freezer overnight, precipitating solid **7b**, which was filtered and rinsed with diethyl ether. Solid **7b** was

collected with 0.16 eq. N,N'-diisopropylurea. (1.8364 g, 4.49 mmol, 45% over two steps). White solid, mp 155-156 °C; ^{1}H NMR (500 MHz, DMSO) δ 10.13 (s, 1H), 9.58 (s, 1H), 8.71 (s, 1H), 7.95 (s, 1H), 7.45 (d, *J* = 7.9 Hz, 1H), 7.25 (t, *J* = 8.0 Hz, 1H), 7.20 (d, *J* = 7.0 Hz, 1H), 2.56 (t, *J* = 7.1 Hz, 2H), 2.41 (t, *J* = 7.1 Hz, 2H), 1.39 (s, 9H); ^{13}C NMR (125 MHz, DMSO) δ 171.00, 170.61, 155.30, 140.91, 130.78, 125.56, 121.59, 121.21, 117.62, 79.05, 31.21, 28.12, 28.01; HRMS (m/z): MS (m/z): $[M]^+$ calc. for $C_{15}H_{20}BrN_3O_4$, 385.1; found 385.1.

7d, tert-butyl 2-(4-((4-hydroxyphenyl)amino)-4-oxobutanoyl)hydrazinecarboxylate

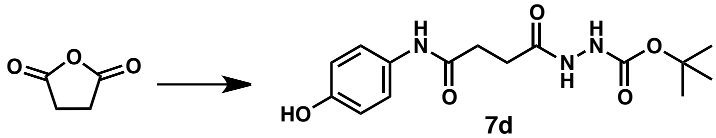

7d

Synthesis followed that of **7a**, with 1.008 g (10.0 mmol) succinic anhydride and with 4-aminophenol in place of 4-bromoaniline. Pale purple solid (1.150 g, 4.98 mmol, 50%) mp 162-164 °C, ^{1}H NMR (500 MHz, DMSO) δ 9.68 (s, 1H), 9.55 (s, 1H), 9.13 (s, 1H), 8.70 (s, 1H), 7.34 (d, *J* = 8.6 Hz, 2H), 6.66 (d, *J* = 8.5 Hz, 2H), 2.48 (s, 2H), 2.38 (t, *J* = 7.2 Hz, 2H), 1.39 (s, 9H); ^{13}C NMR (125 MHz, DMSO) δ 169.30, 156.79, 155.27, 153.04, 131.04, 120.65, 114.98, 78.99, 31.09, 28.39, 28.09. MS (m/z): $[M]^+$ calc. for C15H21N3O5, 323.2; found 323.3.

7e, tert-butyl 2-(4-((3-hydroxyphenyl)amino)-4-oxobutanoyl)hydrazinecarboxylate

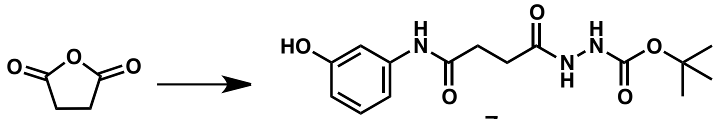

7e

Synthesis followed that of **7b**, with 1.010 g (10.1 mmol) succinic anhydride and 3-aminophenol in place of 3-bromoaniline. Beige solid (894.6 mg, 4.31 mmol, 43%), mp 170-173 °C; ^{1}H NMR (500 MHz, DMSO) δ 9.83 (s, 1H), 9.58 (s, 1H), 9.35 (s, 1H), 7.16 (s, 1H), 7.03 (d, *J* = 8.0 Hz, 1H), 6.93 (d, *J* = 7.9 Hz, 1H), 6.40 (dd, *J* = 8.0, 2.3 Hz, 1H), 2.53 (d, *J* = 7.5 Hz, 2H), 2.39 (t, *J* = 7.5 Hz, 2H), 1.39 (s, 9H); ^{13}C NMR (125 MHz, DMSO) δ 171.14, 170.03, 157.61, 155.34, 140.41, 129.36, 110.08, 109.71, 106.09, 79.06, 31.28, 28.24, 28.14; MS (m/z): [M]+ calc. for $C_{15}H_{21}N_3O_5$, 323.2; found 323.3.

7f, tert-butyl 2-(4-((3-hydroxyphenyl)amino)-4-oxobutanoyl)hydrazinecarboxylate

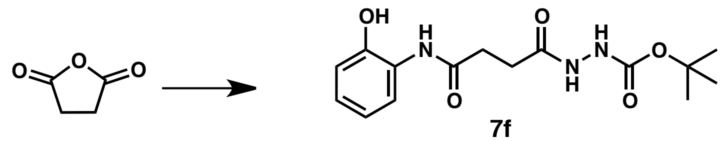

7f

Synthesis followed that of **7b**, with 9.940 mg (0.99 mmol) succinic anhydride and 2-aminophenol in place of 3-bromoaniline. Beige solid (1.37 g, 4.155 mmol, 42% over two steps), mp 157-159 °C; ^{1}H NMR (500 MHz, DMSO) δ 9.73 (s, 1H), 9.58 (s, 1H), 9.31 (s, 1H), 8.73 (s, 1H), 7.69 (d, J = 7.2 Hz, 1H), 6.92 (t, J = 7.6 Hz, 1H), 6.84 (d, J = 7.6 Hz, 1H), 6.74 (t, J = 7.6 Hz, 1H), 2.62 (t, J = 7.1 Hz, 2H), 2.40 (t, J = 7.1 Hz, 2H), 1.39 (s, 9H); ^{13}C NMR (125 MHz, DMSO) δ 171.08, 170.71, 155.28, 147.88, 126.37, 124.53, 122.28, 118.92, 115.74, 79.04, 30.91, 28.40, 28.11; MS (m/z): $[M]^+$ calc. for $C_{15}H_{21}N_3O_5$, 323.2; found 323.3.

7g, tert-butyl 2-(4-((4-hydroxyphenyl)amino)-4-oxobutanoyl)hydrazinecarboxylate

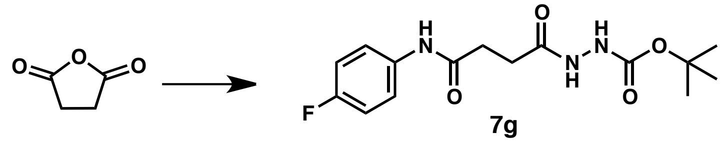

7g

Synthesis followed that of **7b**, with 1.001 g (10.0 mmol) succinic anhydride and 3-aminophenol in place of 3-bromoaniline. White solid (859.7 mg, 8.12 mmol , 81% over two steps), mp 182-190 °C; ^{1}H NMR (500 MHz, DMSO) δ 10.04 (s, 1H), 9.59 (s, 1H), 8.73 (s, 1H), 7.59 (dd, J = 9.0, 5.1 Hz, 2H), 7.12 (t, J = 8.8 Hz, 2H), 2.54 (t, J = 7.3 Hz, 2H), 2.40 (t, J = 7.0 Hz, 2H), 1.38 (s, 9H); ^{13}C NMR (125 MHz, DMSO) δ 171.06, 170.03, 158.71, 155.30, 135.78, 135.76, 120.52, 115.35, 79.03, 31.11, 28.16, 28.11; HRMS (m/z): $[M]^+$ calc. for $C_{15}H_{20}FN_3O_4$, 325.1; found 325.1.

15a, *tert*-butyl 2-(5-((4-bromophenyl)amino)-5-oxopentanoyl)hydrazinecarboxylate.

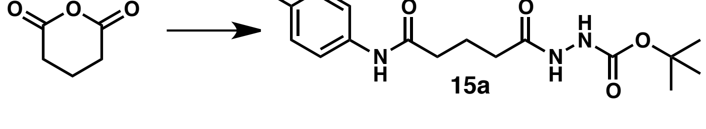

15a

Synthesis followed that of **7a** but with glutaric anhydride 1.141 g (10.0 mmol) in place of succinic anhydride. **15a** was collected with 1.5 eq. N,N'-diisopropylurea (3.08 g, 5.00 mmol, 50% over two steps), mp 161-163 °C; ^{1}H NMR (500 MHz, DMSO) δ 1.39 (s, 10H), 2.12 (t, J = 7.4 Hz, 2H), 10.05 (s, 1H), 9.53 (s, 1H), 8.71 (s, 1H), 7.57 (d, J = 9.0 Hz, 2H), 7.46 (d, J = 8.9 Hz, 2H), 5.50 (d, J = 7.8 Hz, 0H), 2.34 (t, J = 7.5 Hz, 2H), 1.80 (p, J = 7.2 Hz, 2H); ^{13}C NMR

(125 MHz, DMSO) δ 171.46, 170.70, 158.77, 155.37, 135.74, 135.72, 121.04, 115.26, 79.07, 35.47, 32.43, 28.12, 20.92; HRMS (m/z): $[M]^+$ calc. for $C_{16}H_{22}BrN_3O_4$, 399.07937; found 399.07794.

15g, *tert*-butyl 2-(5-((4-fluorophenyl)amino)-5-oxopentanoyl)hydrazinecarboxylate

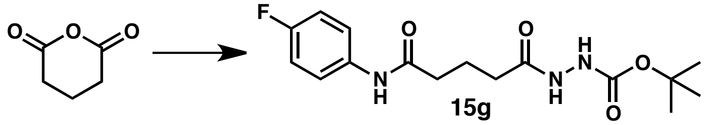

Synthesis followed that of **7a** but with glutaric anhydride (1.1414 g, 10.00 mmol) in place of succinic anhydride and 4-fluoroaniline (1.1520 g, 10.37 mmol) in place of 4-bromoaniline. **15g** was collected with 1.5 eq. N,N'-diisopropylurea (2.9579 g, 5.32 mmol, 53% over two steps), mp 151-156 °C; ^{1}H NMR (500 MHz, DMSO) δ 9.94 (s, 1H), 9.51 (s, 1H), 8.69 (s, 1H), 7.60 (m, 2H), 7.12 (t, *J* = 8.4 Hz, 2H), 2.32 (t, *J* = 7.3 Hz, 2H), 2.12 (t, *J* = 7.3 Hz, 2H), 1.80 (p, *J* = 7.3 Hz, 2H); ^{13}C NMR (125 MHz, DMSO) δ 171.46, 170.70, 158.77, 155.37, 135.74, 135.72, 120.72, 115.34, 115.16, 79.07, 35.47, 32.43, 28.12, 20.92; HRMS (m/z): $[M]^+$ calc. for $C_{16}H_{22}FN_3O_4$, 339.15943; found 339.15906.
